# Wildland-Urban Interface Fires: Toxic Physicochemical Properties of Emitted Particulate Matter and Impacts on Lung Macrophages

**DOI:** 10.1021/acs.est.5c16340

**Published:** 2026-04-01

**Authors:** Glen M. DeLoid, Lila Bazina, Leonardo Calderon, Georgios A. Kelesidis, Jose Guillermo Cedeno Laurent, Irini Tsiodra, Nikolas Mihalopoulos, Luke Fritzky, Nachiket Vaze, Shuo Xiao, Audrey Gaskins, Philip Demokritou

**Affiliations:** † Nanoscience and Advanced Materials Center, Environmental and Occupational Health Sciences Institute (EOHSI), 1857 Rutgers University, Piscataway, New Jersey 08854, United States; ‡ Department of Environmental Occupational Health and Justice, School of Public Health, Rutgers University, Piscataway, New Jersey 08854, United States; § Faculty of Aerospace Engineering, Delft University of Technology, Delft 2629 HS, The Netherlands; ∥ Institute for Environmental Research and Sustainable Development, 54571 National Observatory of Athens, Athens 15236, Greece; ⊥ Environmental Chemical Processes Laboratory, Department of Chemistry, University of Crete, Heraklion 71003, Greece; # New Jersey Medical School, Cancer Institute of New Jersey, 12286 Rutgers University, Newark, New Jersey 07103, United States; ∇ Department of Pharmacology and Toxicology, Ernest Mario School of Pharmacy, 242612 Rutgers University, Piscataway, New Jersey 08854, United States; ○ Department of Epidemiology, Rollins School of Public Health, Emory University, Atlanta Georgia 30322, United States

**Keywords:** wildfire particulate matter, wildland urban interface fire, THP-1 macrophages, innate immune function, polycyclic aromatic hydrocarbons

## Abstract

Due to the growth of urban areas in close proximity to wildlands, wildfires increasingly burn both biomass and man-made materials. The physicochemical properties of emitted particulate matter (PM) from such “wildland-urban interface (WUI)” fires may differ substantially from those of wildland fires and other ambient PM sources. However, the associations between properties and hazards of WUI fire PM have not been studied. Here, we employed a wildfire simulator (WiFS) to reproduce biomass and WUI fires by combusting pinewood and a simplistic WUI fire model (1:1 mixture of pinewood and polyethylene), respectively. WUI fire PM contained high concentrations of the highly toxic and carcinogenic PAH benzo­[c]­fluorene and significant amounts of highly bioactive alkyl and oxygenated PAHs, which were both absent in biomass fire PM, and had a carcinogenicity potential (benzo­[a]­pyrene equivalents, BaP_Eq_) 20 times higher than biomass fire PM. Additionally, exposure of THP-1 macrophages to WUI fire PM, but not biomass fire PM, caused significant reductions in viability and mitochondrial potential, significantly decreased phagocytosis of 1 μm beads, and substantial dysregulation of gene expression. These findings suggest that WUI fire PM exposure may be more hazardous than wildland fire PM exposure, likely due to differences in their chemical profiles.

## Graphical abstract

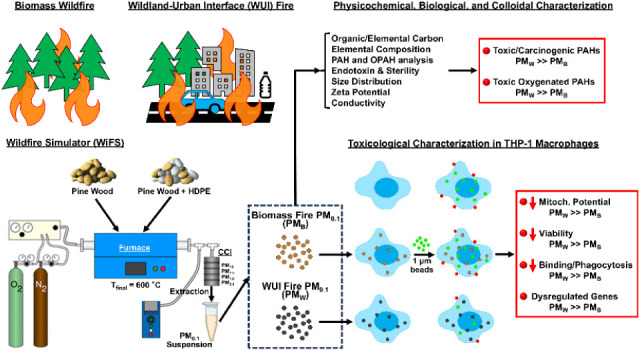

## Introduction

Wildfires have been increasing in frequency, extent, and intensity for four decades and are expected to continue to escalate.^1–3^ In the US each year, over 63,000 wildfires burn an average of 7 million acres of land.^4^ In 2020, about 70% of the California (CA) population experienced more than 100 days of unhealthy air quality with high levels of particulate matter <2.5 μm (PM_2.5_).^5^ During the 2020 wildfires in CA, daily PM_2.5_ reached 350–500 μg/m^3^, far above the 24-h limit of 35 μg/m^3^ set by the US EPA.^6^ In June 2023, wildfire smoke from Canadian wildfires drifted into New York City (NYC) and surrounding areas, causing multiple days of poor air quality, with PM_2.5_ reaching >700 μg/m^3^.^7^ In January 2025, fires in Los Angeles burned 38,000 acres of wildland and urban area, constituting one of the largest wildland-urban-interface (WUI) fires in the US.^8^

Studies from our lab and others have shown that wildfire smoke is a complex mixture of particles and gases, with PM in the fine (0.1–2.5 μm) and ultrafine or nanoscale (<0.1 μm) range,^9,10^ which have complex compositions, consisting primarily of organic carbon (OC, >97%) and can contain high concentrations of polycyclic aromatic hydrocarbons (PAHs), as well as plasticizers, flame retardants, industrial solvents, and heavy metals.^10–16^ At similar ambient background PM exposure levels, WFPM was associated with higher risks of respiratory,^17–20^ cardiovascular,^20–22^ and neuropsychiatric diseases,^23^ as well as all-cause mortality.^24,25^ Studies from our group and others have shown that WFPM causes higher levels of oxidative stress and inflammation in the lung, due to the presence of highly oxidative organic compounds, compared to ambient PM.^12,16^

Due to the burning of both biomass and man-made structures and materials, the PM generated by WUI fires may be more toxic than PM from pure wildland (biomass) fires. Because PM from WUI fires is generated from incineration, not only of wildland fuel but also of homes, industrial buildings, furnishings, vehicles, etc., all of which contain plastics and other man-made materials, its composition, properties, and associated toxicity and health hazards are likely to differ from those of PM generated by pure biomass fires. The potentially greater health hazards associated with PM from WUI fires are of increasing concern due to the rapid urbanization occurring across the globe. Approximately 50 million homes in the US are currently located within WUI areas, with a million new homes being built in WUI areas every three years.^26^

Our current understanding of the chemistry of WUI fires and of the environmental fate/transport and potential health impacts of exposure to WUI fire emissions was recently reviewed by the National Academies of Sciences, Engineering, and Medicine,^27^ which found that the area and number of WUI communities are increasing rapidly. The report highlighted and detailed the variety and contributions of home and urban fuel sources in WUI fires and the resulting complex composition of WUI fire emissions.

While there is growing evidence linking WFPM exposures to adverse health outcomes,^28^ the effects specifically of WUI fire PM exposure have not been studied. A recent review comparing the combustion of man-made materials and biomass found that emissions of several toxic chemicals, including the high molecular weight PAHs, benzene, formaldehyde, and others, were orders of magnitude greater from the combustion of building and vehicle materials than from biomass combustion.^29^ It is therefore likely that WUI fire PM is chemically more complex and potentially more toxic than wildland fire PM. It is thus critical to understand the differences in chemical composition, properties, and toxicity between wildland and WUI fire PM. One critical question is how those differences are reflected in the effects on the health and innate immune function of lung macrophages. Because these cells are the primary defense against inhaled pathogens and particles, any decline in their ability to bind, phagocytose, and eliminate these threats could heighten the risk or severity of respiratory infections.

In the present study, the effects of PM produced by the incineration of either pine wood (PW) alone or a 50:50 mixture of PW and high-density polyethylene (HDPE) on the health and innate immune function of human THP-1 macrophages were investigated. Although real-world WUI fire PM can originate from a wide range of biomass and man-made fuels under complex and varied incineration conditions, the relatively simplistic laboratory incineration of a common wildland biomass fuel (pine wood) and one of the most abundant plastics (PE) provides PM representing the combustion of two of the most likely fuels in such fires.

## Materials and Methods

An overview of the study is shown in Figure 1. Pinewood chips or a 1:1 mixture of pinewood and high-density polyethylene (HDPE) pellets were incinerated using our WildFire Simulator (WiFS)^10,30–33^ to generate PM simulating that created by a pure biomass wildfire and a WUI fire, respectively. The PM generated was size-fractionated using the Harvard Compact Cascade Impactor (CCI)^34^ to collect the PM_0.1_ size fractions of each, hereafter referred to as PM_B_ (PM_0.1_ fraction of particles from a simulated biomass wildfire) and PM_W_ (PM_0.1_ fraction of particles from a simulated WUI fire). Real-time monitoring of particle size, concentration, and gas emissions was conducted throughout incineration, and offline physicochemical characterization of PM was conducted using state-of-the-art analytical methods as described in our previous publications.^10,30–33^

**Figure 1:**
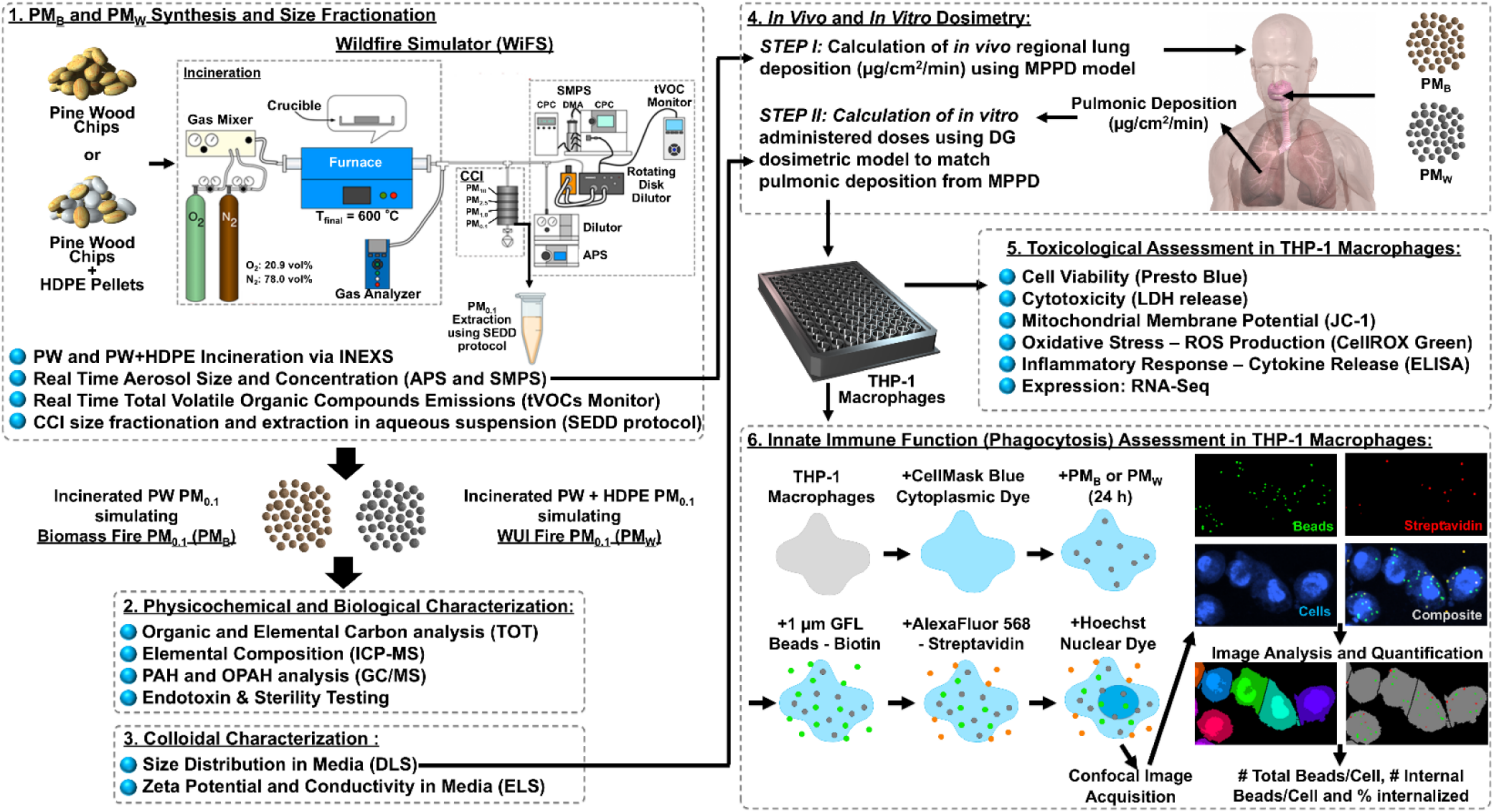
Study design overview.

The Multiple Path Particle Dosimetry (MPPD) model was used to calculate the rate of mass deposition of PM_B_ and PM_W_ (μg/cm^2^/min) in the pulmonary region of a human lung at an ambient concentration of 375 μg/m^3^, which is consistent with levels measured during previous wildfire events.^9,35,36^ The deposition rates were used to determine the total deposition that would occur during 0.5, 5, and 50 day exposures. The distorted grid (DG) in vitro dosimetry model, previously developed by our lab,^37^ was then used to determine administered concentrations of PM_B_ and PM_W_ in cell culture media that would result in 24 h mass depositions (μg/cm^2^) (i.e., doses delivered to cells) corresponding to the 0.5, 5, and 50 day pulmonary mass depositions.

THP-1 macrophages were exposed to suspensions of PM_B_ and PM_W_ at these concentrations for 24 h, and toxicological assessment was conducted, including the evaluation of effects on cell viability, cytotoxicity, mitochondrial membrane potential, oxidative stress, cytokine/chemokine release, and gene expression. Finally, the effects of PM_B_ and PM_W_ exposure on THP-1 macrophage innate immune function were assessed by quantifying the binding and phagocytosis of unopsonized 1 μm fluorescent polystyrene beads.

### Generation, Collection, Size-Fractionation, and Extraction of Simulated Pure Biomass Wildfire PM_0.1_ (PM_B_) and WUI Fire PM_0.1_ (PM_W_)

The PM_0.1_ (≤0.1 μm) size fractions of simulated pure biomass wildfire PM (PM_B_) and WUI fire PM (PM_W_) were synthesized using our WildFire Simulator (WiFS), previously described by the authors.^10,30–33^ Details are provided in Supplementary Methods.

### Real-Time Monitoring of WiFS-Emitted Gases

During combustion of PW or PW + HDPE, carbon monoxide (CO) and total volatile organic compounds (TVOCs) were continuously monitored over time, and the concentrations were recorded. Details are provided in Supplementary Methods.

### Real-Time Monitoring and Physiochemical Characterization of Emitted PM

Details of real-time monitoring and physicochemical characterization of emitted PM are provided in Supplementary Methods.

### Organic and Elemental Carbon Analysis of PM_B_ and PM_W_ from Peak Phase

The analysis of organic and elemental carbon (EC-OC) was performed on PM_B_ and PM_W_ collected on quartz filters during the peak phase. Details are provided in Supplementary Methods.

### Inorganic Elemental Analysis of PM_B_ and PM_W_ Samples

Details of methods for inorganic elemental analysis of PM are provided in Supplementary Methods.

### PAH and OPAH Analysis of PM_B_ and PM_W_ from Peak Phase

The protocol described in Tsiodra et al.^38^ was used to perform quantitative analysis of polyaromatic hydrocarbons (PAHs) and oxygenated PAHs (OPAHs) in the PM_B_ and PM_W_ fractions from the peak phase of combustion. Details of the method are provided in Supplementary Methods.

### Calculation of Carcinogenic Potential from PAH Composition of PM_B_, PM_W_, and PM_HDPE_

The carcinogenic potentials of a mixture of PAHs are expressed as the benzo­[a]­pyrene (BaP) equivalent of the mixture, which is calculated as the sum of the products of the concentration of each PAH and its corresponding toxic equivalency factor (TEF), which is its carcinogenic potency relative to BaP.

### Endotoxin and Microbiological Sterility Testing of PM_B_ and PM_W_ Samples

Endotoxin levels in PMB and PMW were assessed as previously described.^20^ Details are provided in Supplementary Methods.

### Preparation and Colloidal Characterization PM_B_ and PM_W_ Suspensions

Preparation and colloidal characterization of PM_B_ and PM_W_ dispersions were carried out as previously described by the authors.^39–41^ Details are provided in Supplementary Methods.

### Pulmonary Deposition Modeling of PM_B_ and PM_W_ Using the Multiple-Path Particle Dosimetry (MPPD) Model

The Multiple-Path Particle Dosimetry (MPPD) model (v3.04) was used to estimate the rate of mass deposition of PM_B_ and PM_W_ per unit lung surface area (μg/cm^2^/min) in the human pulmonary region as a function of exposure time at an ambient concentration of 375 μg/m^3^. The MPPD results (μg/cm^2^) were then used to determine administered concentrations (μg/cm^3^) that would result in equivalent doses delivered to cells (μg/cm^2^) in in vitro studies, as described below. Additional details on the MPPD modeling approach are provided in Supplementary Methods.

### Calculation of Administered PM_B_ and PM_W_ In Vitro Doses to Match MPPD Deposition Doses Using the Distorted Grid (DG) Dosimetry Model

Administered concentrations of PM_B_ and PM_W_ were determined using the distorted grid (DG) in vitro dosimetry model^37^ to achieve cell-delivered doses (μg/cm^2^) equivalent to pulmonary depositions predicted by the MPPD model for 0.5, 5, and 50 days of exposure at 375 μg/m^3^. Details are provided in Supplementary Methods.

### Culture and Preparation of THP-1 Macrophages

Details of culture and preparation of THP-1 macrophages are provided in Supplementary Methods.

### Exposure of THP-1 Macrophages to PM_B_ and PM_W_

Suspensions of PM_B_ and PM_W_ in RPMI media (with supplements as detailed above) were prepared at concentrations that would result in 24 h mass depositions (μg/cm^2^) (i.e., doses delivered to cells) corresponding to pulmonary mass depositions that would occur in the human lung after 0.5, 5, and 50 day exposures at ambient PM_B_ and PM_W_ of 375 μg/m^3^, which were calculated using the MPPD and DG models, as described above. Culture media was aspirated from mature THP-1 macrophages prepared in 96-well plates; cells were washed once with 200 μL of PBS, and 200 μL of either PM_B_ or PM_W_ suspension at each concentration, vehicle control, or fresh media was dispensed in each well. Plates were then incubated for either 4 h (for oxidative stress assessment, see below) or 24 h (all other assessments) at 37 °C with 5% CO_2_.

### Evaluation of Cell Membrane Integrity (LDH Release)

Methods for evaluation of membrane integrity are provided in Supplementary Methods.

### Evaluation of Cell Viability

Methods for evaluation of cell viability are provided in Supplementary Methods.

### Evaluation of Oxidative Stress (Reactive Oxygen Species Production)

Methods for evaluation of cell viability are provided in the Supplementary Methods.

### Evaluation of Mitochondrial Membrane Potential

Methods for evaluation of mitochondrial membrane potential are provided in Supplementary Methods.

### Assessment of Cytokine/Chemokine Release

Methods for assessment of cytokine/chemokine release are provided in Supplementary Methods.

### Assessment of Innate Immune Function in THP-1 Macrophages

Innate immune function was assessed using methods previously developed by authors to quantify binding and phagocytosis of 1 μm beads.^42,43^ Details of the method are provided in Supplementary Methods.

### Expression Analysis by RNA-Seq

Methods for RNA-seq analysis are provided in Supplementary Methods.

### Statistical Analysis

Toxicity studies were conducted in triplicate for each treatment. Statistical analysis was performed, and graphs for figures were generated using Prism 10 software for Windows (GraphPad Software, Inc., San Diego, CA). The toxicity results were evaluated using paired *t*-tests for comparing vehicle and positive controls with untreated and one-way ANOVA with Tukey’s multiple comparison test for comparing vehicle control with PM_B_ and PM_W_ exposures at each dose.

## Results and Discussion

### Physicochemical Characterization of PM_B_ and PM_W_ and Other Gaseous Copollutants

The elemental compositions of PM_B_ and PM_W_ differed substantially (Figure S1). Although sulfur was the predominant species in both PM_B_ and PM_W_, it made up a much greater percentage of PM_W_ (75.52%) than of PM_B_ (43.14%). Other than sulfur, the three most prominent components of PM_B_ were calcium (17.52%), sodium (11.67%), and aluminum (8.74%), while the other most prominent elements in PM_W_ were calcium (4.55%), aluminum (4.47%), potassium (3.96%), and iron (3.88%).

Analysis of PAHs revealed striking differences between PM_B_ and PM_W_ (Figure 2). Whereas retene (a marker of biomass fires) made up the majority of PM_B_ PAHs (78.01%), which also included pyrene (7.59%), fluoranthene (5.95%), phenanthrene (3.24%), and chrysene (1.17%) (Figure 2A,B), retene comprised only 38.57% of PM_W_ PAHs, which also included higher concentrations of pyrene (9.43%), phenanthrene (9.40%), and fluoranthene (9.14%), as well as benzo­(b+c)­fluorene, which was not present in PM_B_ (Figure 2A,C). The presence of benzo­(b+c)­fluorene (which includes 11H-benzo­(b)­fluorene and 7H-benzo­(c)­fluorene) suggests greater potential toxicity and carcinogenicity of PM_W_, since 7H-benzo­(c)­fluorene is known to be highly toxic and carcinogenic.^44,45^ However, the individual contributions of 7H-benzo­(c)­fluorene and 11H-benzo­(b)­fluorene to the benzo­(b+c)­fluorene component could not be determined because both species elute together in a single combined spectra peak.^38^ Based on PAH analysis of PM_0.1_ from incinerated HDPE alone (PM_HDPE_) (Figure 2A,D), the PAH profile of PM_W_ does not appear to be an equally weighted mixture of PM_B_ and PM_HDPE_ but instead maintains a relatively high percentage of retene and contains considerably lower amounts of other PAHs than PM_HDPE_. This suggests that the pinewood (biomass) component contributes an overall greater portion of PAHs to PM_W_ than the HDPE component. In addition, PM_W_ and PM_HDPE_ contained significant amounts of oxygenated PAHs, in contrast to PM_B_, in which OPAHs were not detected (Figure 3).

**Figure 2:**
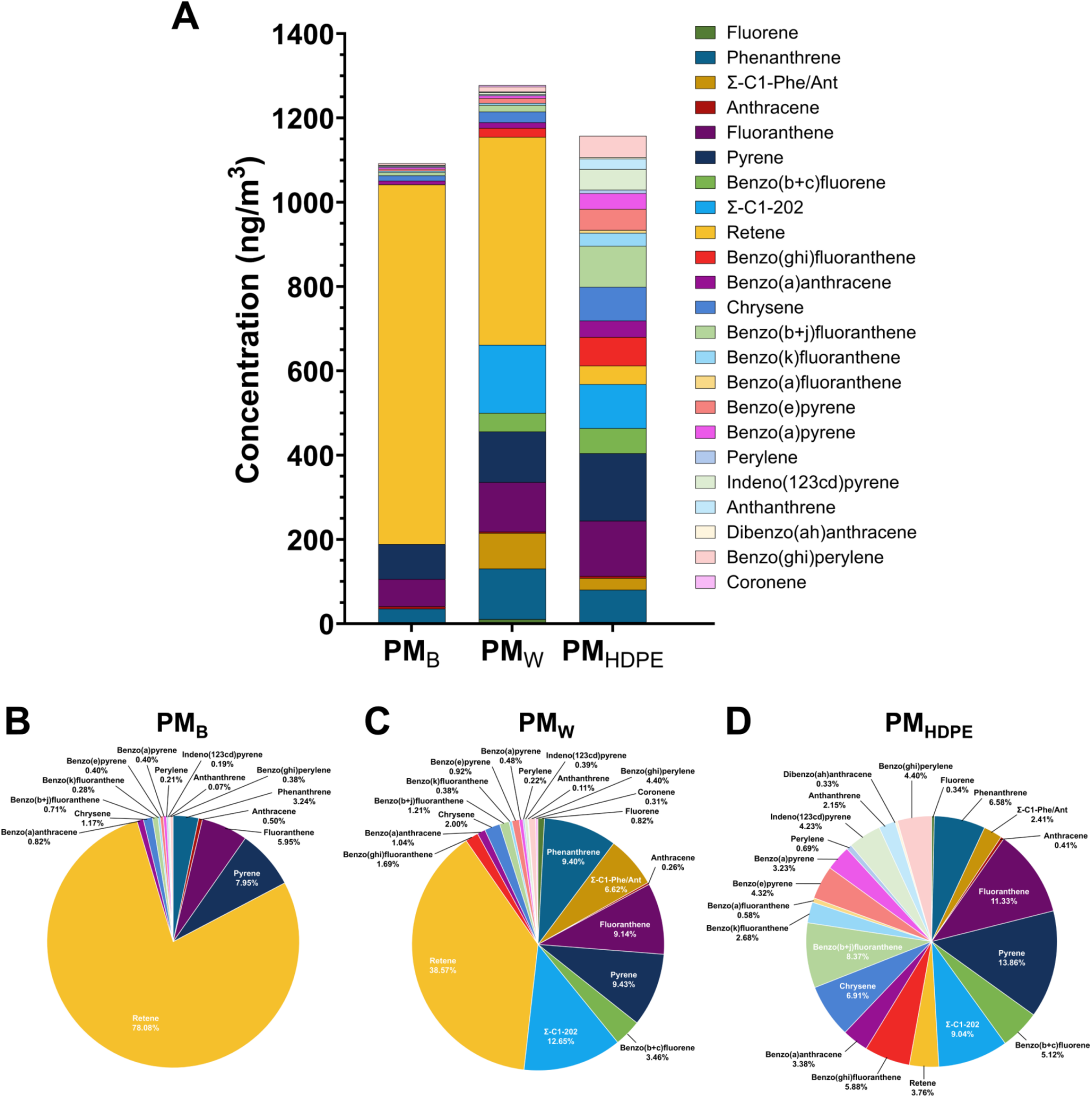
PAH concentration and percentage composition profiles of PM_B_, PM_W_, and PM_HDPE_. A. Concentrations of PAHs in PMB, PMW, and PMHDPE. B. Pie chart of PAHs in PMB. C. Pie chart of PAHs in PMW. D. Pie chart of PAHs in PMHDPE.

**Figure 3:**
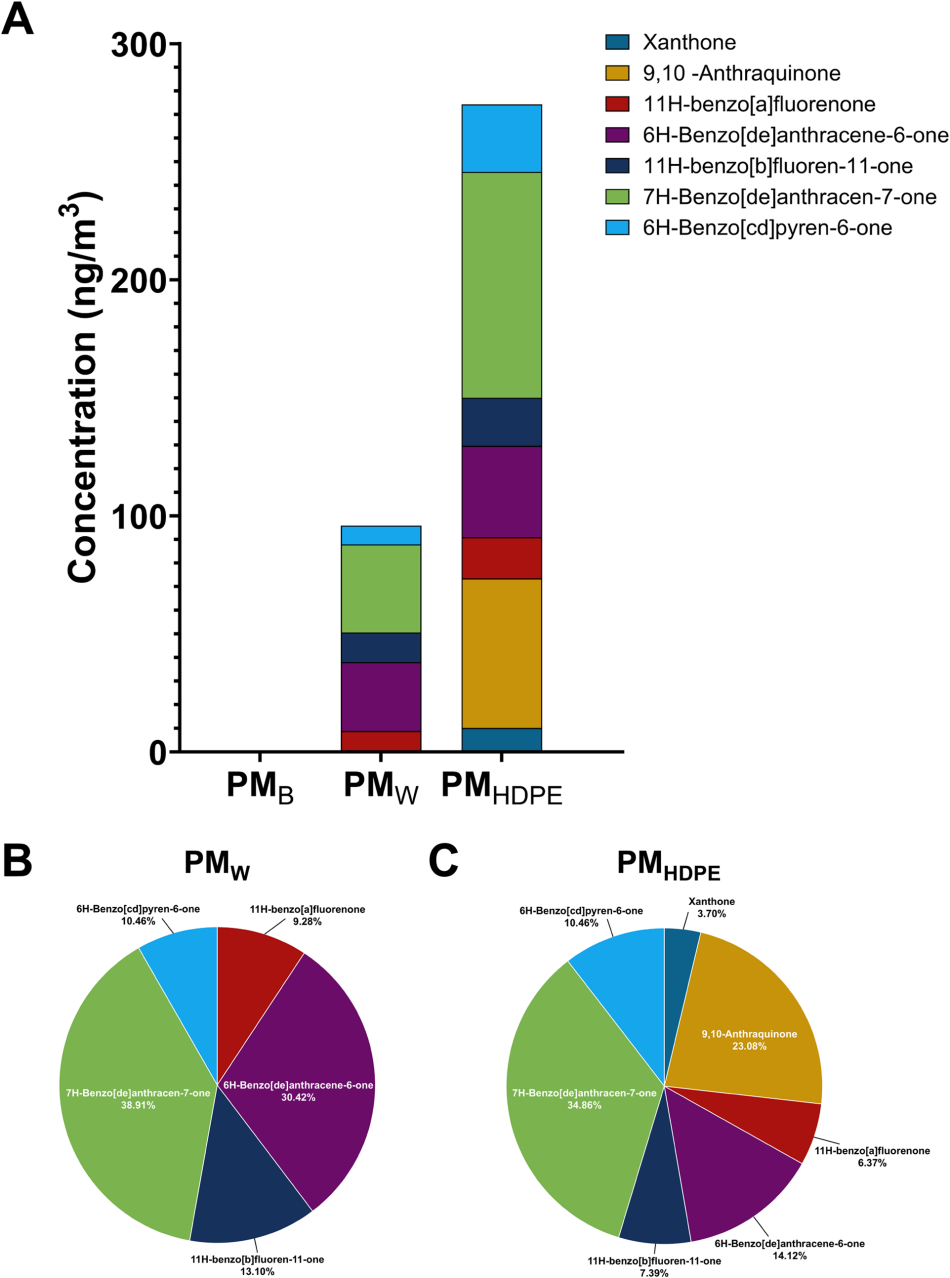
Oxygenated PAH concentration and percentage composition profiles of PM_B_, PM_W_, and PM_HDPE_. A. Concentrations of oxygenated PAHs in PMB, PMW, and PMHDPE. B. Pie chart of oxygenated PAHs in PMW. C. Pie chart of oxygenated PAHs in PMHDPE.

PAH composition profiles by PAH category (Legacy—the original 16 PAHs listed by the EPA, Emerging—more recently discovered, alkylated, and oxygenated) revealed that whereas PM_B_ contained substantially greater amounts of emerging PAHs (860 ng/m^3^) than either PM_W_ (579 ng/m^3^) or PM_HDPE_ (260 ng/m^3^), both PM_W_ and PM_HDPE_ contained considerable amounts of alkyl and oxygenated PAHs, whereas PM_B_ contained none of either (Figure S2). Specifically, PM_W_ contained 246 ng/m^3^ alkyl and 96 ng/m^3^ oxygenated PAHs, and PM_HDPE_ contained 132 ng/m^3^ alkyl PAHs and 274 ng/m^3^ oxygenated PAHs. Since oxygenated PAHs are generally known to be highly toxic, this suggests a greater potential toxicity for WUI fire PM (PM_W_) than for pure biomass wildfire PM (PM_B_).

The PAH and OPAH profiles of PM_W_ contained multiple species that were not present in PM_B_, including fluorene, C1-alkylated phenanthrenes and anthracenes, benzo­(b+c)­fluorene, benzo­(ghi)­fluoranthene, coronene, 11H-benzo­[a]­fluorenone, 6H-benzo­[de]­anthracene-6-one, 11H-benzo­[b]­fluoren-11-one, 7H-benzo­[de]­anthracen-7-one, and 6H-benzo­[cd]­pyren-6-one (Figures 2 and 3). With the exception of coronene, all of these species were also present in PM from the incineration of HDPE alone (PM_HDPE_). The presence of coronene in PM_W_ alone may thus represent the synergistic generation of a unique chemical product during cocombustion of pinewood and HDPE. Further studies of PM generated by combustion of a wider range and different mixtures of potential WUI fuels are needed to determine whether coronene or other chemical signatures might be useful specific markers of WUI fire PM.

Results of real-time monitoring of emissions from the incineration of pinewood (PW) and PW+HDPE, with particle number concentrations measured by SMPS and APS as a function of time and temperature during incineration, are shown in Figures S3 and S4, respectively. The maximum particle concentrations, with diameters ranging from 5.52 to 209.1 nm, measured by SMPS were observed approximately 22 min after combustion at around 500 °C, yielding a particle number concentration of ∼1 × 10^8^ particles/cm^3^ for PW and ∼2 × 10^8^ particles/cm^3^ for PW+HDPE. Particles with larger diameter sizes (>523 nm) acquired by APS similarly had the highest particle concentration after approximately 20 min of combustion, resulting in a particle concentration of ∼6 × 10^5^ particles/cm^3^ for PW and ∼6.1 × 10^5^ particles/cm^3^ for PW+HDPE.

The aerosol mass size distributions and concentrations of each size fraction during the incineration of PW and PW+HDPE are shown in Figure S5. As expected, PM_0.1_ had one of the highest mass concentrations for both PW and PW+HDPE, at approximately 21 mg/m^3^ for PW PM and 15 mg/m^3^ for PW+HDPE PM. The PM_0.1–2.5_ mass concentration was somewhat higher, at 31 mg/m^3^ for PW PM and 16 mg/m^3^ for PW+HDPE PM, which is typical for such combustion processes.

Quantification of gaseous volatile organic compounds (VOCs) and carbon monoxide emissions was also acquired as a function of time and temperature throughout the combustion process of HDPE and is represented in Figures S6 and S7. The maximum gas emissions acquired occurred after 25 min of combustion at ∼600 °C, yielding a VOC emission of ∼1.5 × ∼10^4^ parts per billion (ppb) for the incineration of both PW and PW+HDPE and a carbon monoxide emission of ∼700 ppm for PW incineration and ∼900 ppm for PW+HDPE incineration.

The 1:1 pinewood:HDPE fuel mixture was chosen for this study as an initial simplistic starting point for investigating the physicochemical and toxicological differences between pure biomass and WUI fire PM. The fuel composition of real-world WUI fires is likely much more complex and varied, including a mixture of woods, as well as a wide variety of man-made materials, including construction materials, stored petroleum products, industrial and household chemicals, as well as a mixture of plastic polymers. In addition, whereas the pinewood/HDPE mixture in this study was incinerated under flaming conditions, burning of both wildland and man-made fuels in a WUI fire is likely to occur to a significant extent under smoldering conditions, which would likely produce PM with very different chemical compositions and toxicological properties. Smoldering combustion is characterized by slow burning rates without flame at low temperatures (450–700 °C).^46,47^ During solid fuel burning, the release of heat is considerably lower (6–12 kJ/g) than the heat released during flaming combustion (16–30 kJ/g).^48^ Smoldering conditions are observed in outdoor megafires with fuels like peat and coal.^49^ Flaming combustion, on the other hand, produces intense heat initially and gradually weakens and becomes constant.^50^ Particles generated from smoldering fires have been found to be more toxic than those produced from more efficient biomass burning^51,52^ and have specifically been linked to adverse cardiovascular effects.^53^ In future studies, we will employ more varied and complex mixtures of WUI fuels, and both flaming and smoldering conditions, to cover the range of fuels and combustion conditions likely present in real-world WUI fires.

### Endotoxin and Sterility Analysis

After a 14-day incubation period, no microorganisms were detected on agar plates inoculated with PM_B_, PM_W_, or the vehicle control. No endotoxin was found in the PM_B_ or PM_W_ samples or vehicle controls, with all results falling below the Limit of Detection (LOD).

### Characterization of Colloidal Properties of PM_B_ and PM_W_ Suspensions in Water and Cell Culture Media

Results of colloidal characterization of PM_B_ and PM_W_ suspended in both water and RPMI1640 + 10% FBS are presented in Table S1. The critical dispersion sonication energy (DSEcr), which is the sonication energy required to achieve a stable aqueous suspension of particles with the lowest possible degree of agglomeration and thus the smallest possible hydrodynamic diameter (d_H_) for each particle in water, was determined as previously described^40,54^ and found to be 1087.8 J/mL for both PM_B_ and PM_W_. The PM_B_ suspension in water had a hydrodynamic diameter (d_H_) of 421.9 ± 30.5 nm, a polydispersity index (PdI) of 0.199 ± 0.020, and a negative zeta potential (ζ) value of −34.30 ± 0.95 mV, demonstrating an effective electrostatic stabilization of the particles and a favorable colloidal dispersion. The PM_W_ suspension in water had a d_H_ of 238.5 ± 1.2 nm, a PdI of 0.318 ± 0.029, and a ζ value of −27.40 ± 11.80 mV. Both particle suspensions were stable in water for 24 h. In contrast, suspensions of PM_B_ and PM_W_ in cell culture media (RPMI + 10% FBS) exhibited smaller sizes, greater polydispersity, and less negative zeta potentials. The PM_B_ suspension in media had a d_H_ of 59.0 ± 50.5 nm, a PdI of 0.358 ± 0.002, and a ζ value of −9.86 ± 0.71 mV, while the PM_W_ media suspension had a d_H_ of 31.6 ± 12.4 nm, a PdI of 0.561 ± 0.233, and a ζ value of −10.00 ± 0.96 mV. Such increases in zeta potential are anticipated due to adsorption of negatively charged serum proteins and formation of a protein corona.

### Human Lung Deposition of PM_B_ and PM_W_ during Target Exposures

The MPPD model was used to assess the PM_B_ and PM_W_ deposition in the pulmonary region of the lung after 0.5, 5, and 50 days of exposure at an ambient concentration of 375 μg/m^3^, which represent reasonable real world exposure scenarios in regions with frequent wildfire or WUI fire events. The PM mass pulmonary deposition rate (μg/cm^2^/min), deposition over 0.5, 5, and 50 day exposures to PM_B_ and PM_W_ are summarized in Table S2. The rate of deposition for both PM_B_ and PM_W_ in the pulmonary region of the human calculated using the MPPD was 3.26 × 10^–5^ μg/cm^2^/min. The target delivered doses for 0.5, 5, and 50 wildfire days, calculated by multiplying the deposition rate by the exposure durations in seconds, were 1.98 × 10^–3^, 1.98 × 10^–2^, and 0.198 μg/cm^2^, respectively.

### Administered In Vitro Cell Culture PM_B_ and PM_W_ Concentrations Required to Match Human Lung Deposition for Target Exposures

The DG in vitro dosimetry model^37^ was used to determine administered concentrations of PM_B_ and PM_W_ in culture media that would result in 24 h mass depositions (μg/cm^2^) corresponding to pulmonary mass depositions after 0.5, 5, and 50 day wildfire exposures at ambient PM_B_ and PM_W_ of 375 μg/m^3^, calculated using the MPPD model to be 1.98 × 10^–3^, 1.98 × 10^–2^, and 0.198 μg/cm^2^. The administered doses, determined using the DG model, required to match these delivered dose targets were 0.118, 1.180, and 11.80 μg/mL for PM_B_, and 1.394, 13.94, and 139.4 μg/mL for PM_W_ (Table S2).

### Carcinogenic Potential of PM_W_ and PM_B_

The carcinogenic potentials, expressed as Benzo­[a]­pyrene equivalents (BaP_Eq_), of PM_W_ and PM_HDPE_ were considerably higher than that of PM_B_ (Figure S8). Specifically, the BaP_Eq_ values of PM_B_, PM_W_, and PM_HDPE_ were 24, 483, and 799 ng/m^3^, respectively. The majority of the BaP_Eq_ of PM_W_ and PM_HDPE_ was attributable to 7H-benzo­(c)­fluorene (92% and 75%, respectively), calculated assuming that it comprised half of the combined benzo­(b+c)­fluorene peak, which was absent in PM_B_.

### Evaluation of PM_B_ and PM_W_ Impact on Lung Macrophage Toxicity

Results of the in vitro toxicity assessment of PM_B_ and PM_W_ in PMA-differentiated THP-1 macrophages are summarized in Figure 4.

**Figure 4:**
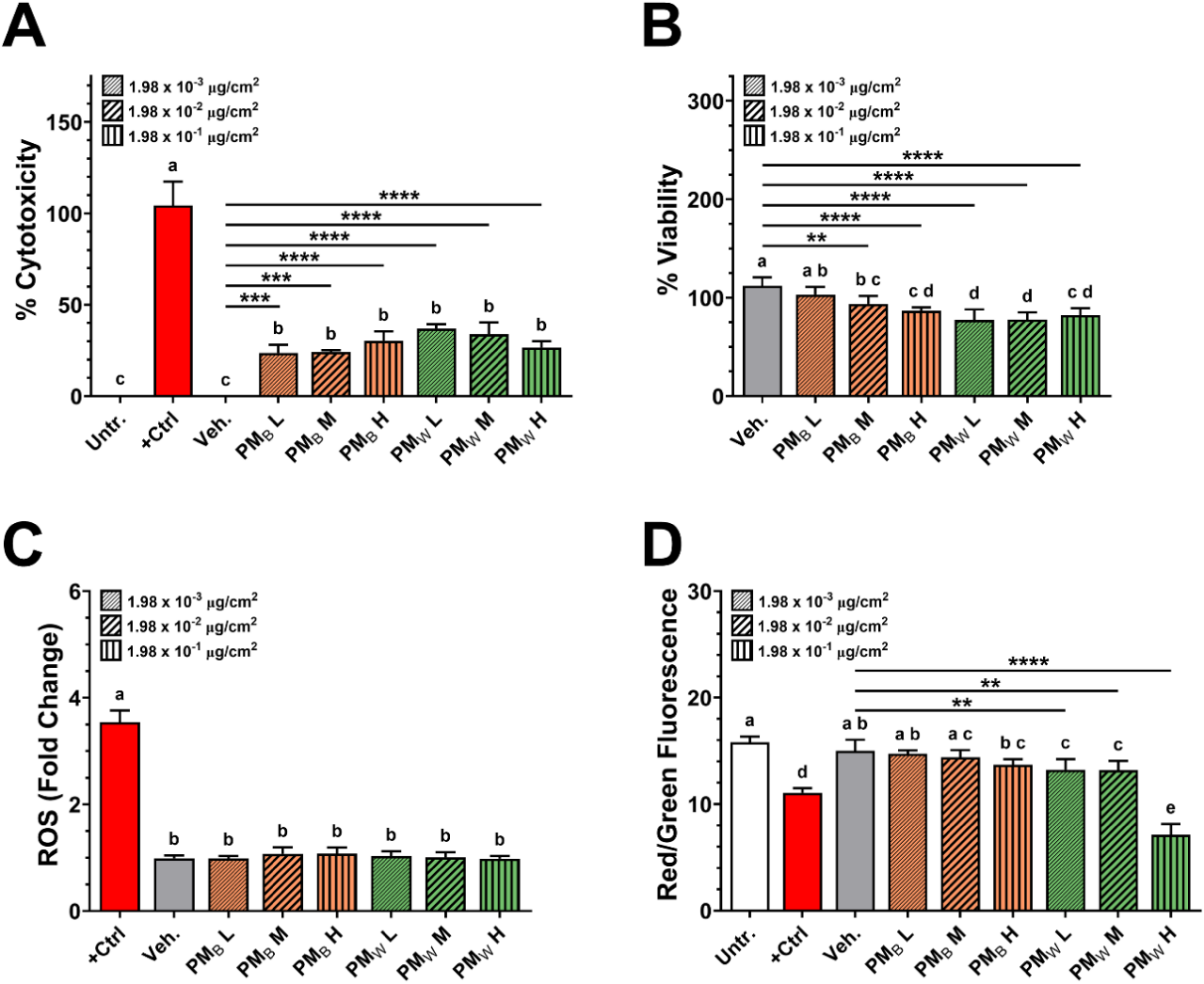
Toxicological effects of PM_B_ and PM_W_ on THP-1 macrophages. **A.** Cytotoxicity (% LDH release) after 24 h exposure to PM_B_, PM_W_, vehicle control, or lysis buffer. **B.** Cell viability, evaluated with the PrestoBlue assay following exposure to PM_B_, PM_W_, or vehicle control for 24 h. **C.** Intracellular reactive oxygen species (ROS) assessed after 4 h exposures to PM_B_, PM_W_, vehicle control, or menadione (positive control). **D.** Mitochondrial potential of macrophages assessed using the JC-1 mitochondrial membrane potential kit after 24 h exposure to PM_B_, PM_W_, vehicle control, or CCCP (positive control) for 24 h. (*N* = 3. ** *p* < 0.01, *** *p* < 0.001, **** *p* < 0.0001).

Exposure to both PM_B_ and PM_W_ for 24 h at all three doses caused significant cytotoxicity, as indicated by LDH release (Figure 4A). Exposure to PM_B_ resulted in ∼25% cytotoxicity at the low and middle doses (*p* < 0.001), and ∼30% cytotoxicity at the high dose (*p* < 0.0001), while PM_W_ exposure resulted in ∼37% toxicity at the low dose (*p* < 0.0001), and somewhat lower (but not significantly different) cytotoxicity at the middle and high doses (*p* < 0.0001). These observations indicate that PM_B_ and PM_W_ at delivered cellular doses as low as 1.98 × 10^–3^ μg/cm^2^ can disrupt the plasma membranes of PMA-differentiated THP-1 macrophages.

Cell viability of PMA-differentiated macrophages following PM_B_ and PM_W_ exposure was evaluated by measuring the mitochondrial enzyme activity (Figure 4B). Only the middle and high doses of PM_B_ caused significant decreases in cell viability, with viability reduced to ∼85% (*p* < 0.01) at the middle delivered dose (1.98 × 10^–2^ μg/cm^2^) and to ∼80% at the high delivered dose (1.98 × 10^–1^ μg/cm^2^). Exposure to PM_W_ caused a significant reduction in viability at all three doses, decreasing to ∼73% (*p* < 0.0001) at the low (1.98 × 10^–3^ μg/cm^2^) and middle (1.98 × 10^–2^ μg/cm^2^) delivered doses, and ∼75% (*p* < 0.0001) at the high (1.98 × 10^–1^ μg/cm^2^) delivered dose.

This greater reduction in viability by PM_W_ compared to PM_B_ may be attributed to the differences in the PAH profiles between PM_B_ and PM_W_, particularly the presence of alkyl and oxygenated PAHs (OPAHs) and emerging PAHs detected in PM_W_ that were absent in PM_B_. OPAHs, and particularly species like 6H-benzo­[cd]­pyren-6-one have mutagenic properties and are connected to tumor promotion.^55^ The presence in PM_W_ of 7H-benzo­[c]­fluorene, an emerging PAH with a toxic equivalent factor (TEF) 20 times higher than benzo­[a]­pyrene (BaP)^56^ and considered a potent lung tumorigen,^57^ may also have contributed to its greater toxicity compared to PM_B_. The presence of this compound even in small quantities can significantly affect the estimated carcinogenicity (BaP_Eq_) of a mixture of PAHs^38^ as seen in Figure S8 and discussed above. Inorganic elements detected in PM_W_ samples (e.g., Fe, S), represented in Figure S1, could also have contributed to the reduction in cellular viability, and the higher percentage of sulfur in PM_W_ could be responsible for part of the greater impact of PM_W_. Previous studies have found that PM containing PAHs and metals from ambient and occupational exposures has genotoxic properties, which could result in DNA damage, leading to cell cycle arrest, apoptosis, and reduced viability.^58,59^

No statistically significant increase in oxidative stress (ROS production) was observed after exposure to any dose of either PM_B_ or PM_W_ compared to untreated and vehicle control (Figure 4C).

Mitochondrial membrane potential was assessed after 24 h exposures to PM_B_ and PM_W_ using the JC-1assay (Figure 4D). Exposure to PM_B_ had no significant effect on the mitochondrial membrane potential at any dose. In contrast, all doses of PM_W_ caused a significant decrease in the mitochondrial membrane potential, indicated by a reduction in the ratio of red to green fluorescence in the JC-1 assay. At the low (1.98 × 10^–3^ μg/cm^2^) and middle (1.98 × 10^–2^ μg/cm^2^) doses, PM_W_ caused a ∼15% reduction in red/green fluorescence (*p* < 0.01), and at the high (1.98 × 10^–1^ μg/cm^2^) delivered dose, PM_W_ exposure reduced the red/green fluorescence ratio by ∼50% (*p* < 0.0001) compared to vehicle control. Treatment with CCCP (positive control) resulted in a ∼30% (*p* < 0.0001) reduction in the red/green fluorescence ratio, as expected.

We previously found that exposure of THP-1 macrophages to PM_0.1_ generated by incineration of HDPE alone (PM_HDPE_) similarly resulted in increased cytotoxicity, reduced viability, and impaired mitochondrial membrane potential, without a significant increase in ROS generation.^60^ This suggests that the combustion products of the HDPE component of the pinewood+HDPE fuel used to produce PM_W_ were likely responsible for most of the observed toxic effects.

It is worth noting that PMW caused a significant decrease in mitochondrial membrane potential, while no ROS production was detected. Usually, a decrease in the mitochondrial membrane potential is closely associated with increased ROS production. Since ROS species are labile, this could be the result of exposure time (4 h used in this study). Although our positive control (menadione) produced a strong ROS signal at 4 h, ROS produced as a result of exposure to PMW may have occurred earlier (or later) or at a slow and steady rate that was neutralized by intracellular antioxidant enzymes and molecules (glutathione) and thus never accumulated to a detectable level. In future studies, a mitochondrial-ROS-specific probe, such as a mitochondrial superoxide assay, will be employed, and the depletion of reduced glutathione or other markers of oxidative stress (lipid peroxidation and protein carbonylation) will be assessed to shed more light on the ROS generation issue.

### Impact of PM_B_ and PM_W_ on Macrophage Cytokine and Chemokine Release

The inflammatory responses in PMA-differentiated THP-1 macrophages after 24 h exposures to the highest dose (1.98 × 10^–1^ μg/cm^2^) of PM_B_ and PM_W_ were assessed by quantitative analysis of 48 cytokines and chemokines in cell supernatants. The results revealed no significant changes in the release of any of the 48 cytokines by either PM_B_ or PM_W_ compared to untreated or vehicle controls (Figure S9). Treatment with LPS (positive control) resulted in a significant increase in multiple cytokines and chemokines, as anticipated. The lack of a cytokine/chemokine response suggests that neither PM_B_ nor PM_W_ elicits a significant inflammatory response.

### Impact of PM_B_ and PM_W_ on Macrophage Phagocytosis

The effect of PM_B_ and PM_W_ exposure on phagocytosis of unopsonized green fluorescent 1.0 μm polystyrene beads was assessed as described in Supplementary Methods. Representative images of untreated, positive control (cytochalasin D), and PM_B_- or PM_W_-treated THP-1 macrophages after incubation with beads and staining, as well as results of quantitative analysis of phagocytosis, are shown in Figure 5. Internalized beads appear green, while external beads appear orange-yellow (due to binding of red fluorescent AlexaFluor568-streptavidin to the green fluorescent biotinylated beads). In untreated cells (Figure 5A) most beads appear to be internalized (green only). In cells treated with cytochalasin D (positive control, inhibitor of actin polymerization; Figure 5B), few internalized (green) beads are seen, and external (orange-yellow) beads appear to be bound and accumulating at the plasma membrane, indicating a defect in internalization, as expected.

**Figure 5:**
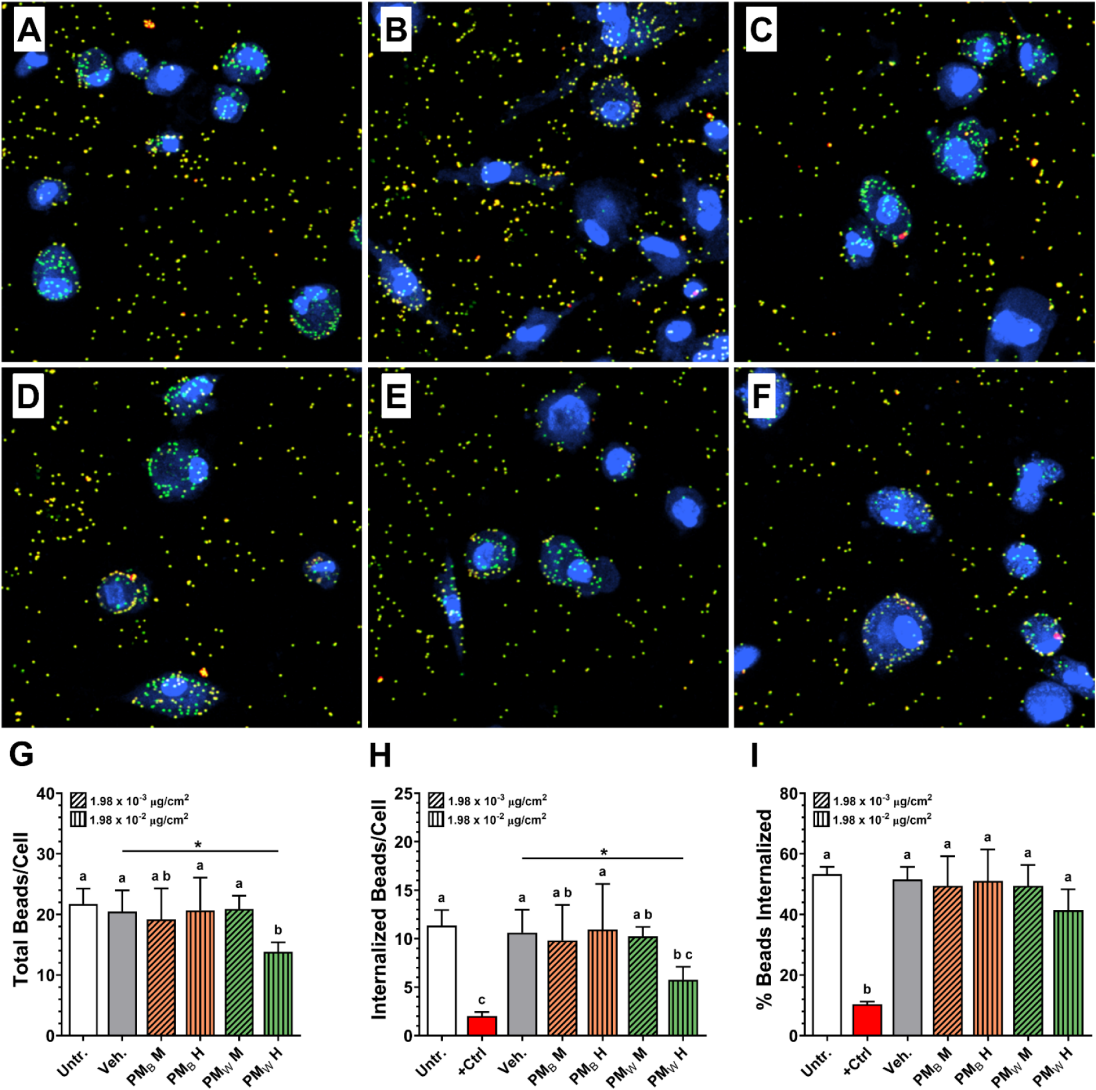
Evaluation of phagocytosis of fluorescently labeled polystyrene beads after exposure to PM_B_ or PM_W_. **A.** Untreated (media only). **B.** Cytochalasin D (positive control). **C.** PM_B_ at 5 d exposure equivalent dose (delivered dose: 1.98 × 10^–2^ μg/cm^2^). **D.** PM_B_ at 50 d exposure equivalent dose (delivered dose: 1.98 × 10^–1^ μg/cm^2^). **E.** PM_W_ at 5 d exposure equivalent dose (delivered dose: 1.98 × 10^–2^ μg/cm^2^). **F.** PM_W_ at 50 d exposure equivalent dose (delivered dose: 1.98 × 10^–1^ μg/cm^2^). **G.** Average total number of beads per cell (internalized + external/bound). **H.** Average number of internalized beads per cell. **I.** Average percentage of beads internalized. Cytochalasin D. (*N* = 3. * *p* < 0.05).

In THP-1 macrophages treated with the middle or high doses of PM_B_, most beads appear to be internalized, similar to the untreated control (Figure 5C,D). Likewise, in cells exposed to PM_W_ at the middle dose, the uptake of beads appears to be unimpaired (Figure 5E). However, at the high dose, THP-1 macrophages exposed to PM_W_ appear to contain fewer internalized (green only) beads (Figure 5F), suggesting a defect in either binding or internalization.

Consistent with the impressions from confocal images, exposure to PM_B_ at the middle and high doses and exposure to PM_W_ at the middle dose had no effect on the total number of beads per cell (Figure 5G), internalized beads per cell (Figure 5H), or the percentage of beads internalized (Figure 5I). However, at the high dose, exposure to PM_W_ caused a significant decrease of ∼50% in both total beads per cell and internalized beads per cell (*p* < 0.05). Because total (bound + internalized) and internalized beads were decreased equally, there was no effect on the percentage of beads internalized. These findings suggest that PM_W_ caused a defect primarily in the binding step of phagocytosis rather than a defect in internalization. This could be a result of either a decrease in expression of phagocytic receptors, such as the scavenger receptor MARCO, or blockade of the same receptors by PM_W_ particles. Because the ability of alveolar macrophages to bind, phagocytose, and neutralize pathogens and particles is critical to innate immune defense in the lung, impairment of those functions by WUI PM could represent a serious health hazard resulting from WUI fires that may be more pronounced than impairment from exposure to PM from a pure biomass wildfire.

Interestingly, in our previous study in which THP-1 macrophages were exposed to PM_0.1_ from incinerated HDPE alone (PM_HDPE_),^60^ while phagocytosis was also significantly reduced, this was accompanied by a corresponding significant reduction in internalization, indicating an impairment of phagocytosis rather than binding.^60^ This discrepancy may be a result of greater amounts of toxic chemical species, particularly PAHs, present in PM_HDPE_ compared to PM_W_. As noted above, the PAH profile of PM_W_, while having greater amounts of high molecular weight and toxic PAH and OPAH species than PM_B_, had an overall profile closer to that of PM_B_ than that of a 50/50 mixture of PM_B_ and PM_HDPE_. Further work is needed with PMW generated with a wider range of wildland and man-made fuels to assess the effects of different fuels on the binding and internalization functions of macrophages.

### Impact of PM_B_ and PM_W_ on Gene Expression in Macrophages

The impact of PM_B_ and PM_W_ exposure on gene expression was assessed by RNA-seq. The differential expression of genes after treatment of THP-1 macrophages with the highest doses of PM_B_ and PM_W_ (equivalent to 50 days of inhalation exposure at 375 μg/m^3^) is summarized in Figure 6. A total of 421 protein-coding genes (PCGs) were uniquely expressed in PM_B_-treated THP-1 macrophages, while 451 PCGs were exclusively expressed by controls, and 12,017 PCGs were expressed by both PM_B_-treated and control cells (Figure 6A). THP-1 macrophages exposed to PM_W_ uniquely expressed 577 PCGs, while untreated controls uniquely expressed 621 genes, and both PM_W_-exposed and control cells expressed 11,850 PCGs (Figure 6B). These findings indicate that exposure to either PM_B_ or PM_W_ initiates expression of several hundred PCGs while repressing the expression of hundreds of others. Notably, treatment with PM_W_ (simulated WUI fire PM) activated or repressed expression of about 25% more PCGs than exposure to PM_B_ (simulated pure wildland/biomass fire), which is further evidence of the likely greater hazards posed by WUI fire PM compared to pure wildland fire PM.

**Figure 6:**
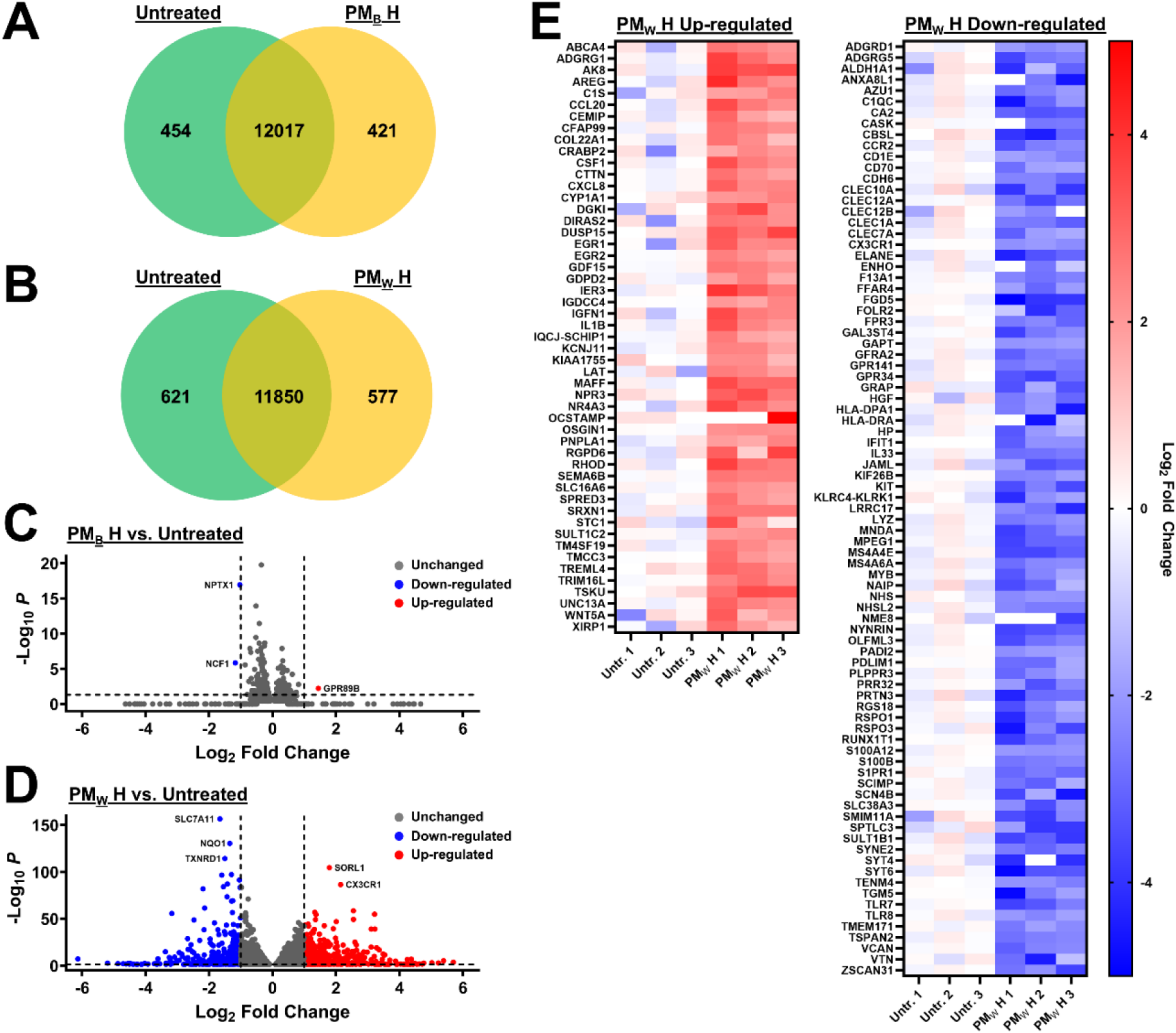
Expression/RNA-seq. **A.** Venn diagram illustrating the number of protein-coding genes uniquely expressed and expressed by both untreated and PM_B_-treated cells. **B.** Venn diagram showing the number of protein-coding genes uniquely expressed and expressed by both untreated and PM_W_-treated cells. **C.** Volcano plot indicating protein-coding genes significantly downregulated (*p* < 0.05, −log_10_ *p* > 1.3) and upregulated in PM_B_-treated macrophages relative to expression in untreated macrophages. **D.** Volcano plot indicating protein-coding genes significantly downregulated (*p* < 0.05, −log_10_ *p* > 1.3) and upregulated in PM_W_-treated macrophages relative to expression in untreated macrophages. **E.** Heat map illustrating significantly downregulated and upregulated genes (*p* < 0.05, −log_10_ *p* > 1.3) with fold change >2 (log_2_ fold change >1) in individual replicate samples of untreated and PM_W_-treated macrophages.

The volcano plots (log_2_ fold change vs −log_10_ *p*), which depict PCGs whose expression was significantly dysregulated (*p* < 0.05, −log_10_ *p* > 1.3), reveal striking differences between the effects of exposure to PM_B_ and PM_W_, with PM_W_ significantly altering expression of far more PCGs than PM_B_ (Figure 6C,D). Whereas PM_B_ significantly upregulated expression of just one PCG and downregulated expression of two, PM_W_ significantly upregulated 444 and downregulated 359 PCGs. PCGs significantly downregulated or upregulated with a fold change >2 (log_2_ fold change >1) are illustrated in the expression heatmaps (Figure 6E). There were no genes significantly upregulated or downregulated with a fold change greater than 2 by exposure to PM_B_.

Several genes involved in immune responses, detoxification, and cell death were significantly upregulated (−log_10_ *p* > 1.3, log_2_ fold change >1) by exposure to PM_W_ (but not PM_B_). Interleukin 8 (CXCL8, IL-8), a pro-inflammatory chemokine, was significantly upregulated. In an intact lung, increased expression of IL-8 would induce or exacerbate cellular infiltration and inflammation. Interleukin-1 beta (IL1B), a key pro-inflammatory cytokine, was also significantly upregulated by PM_W_ (but not PM_B_). Although increased expression of IL1B could lead to induction or exacerbation of inflammation in the lung, its transcript is produced as a preprotein, which requires activation by inflammasomes (e.g., NALP3), which form in response to a variety of noxious stimuli, to cleave the preprotein to produce the active IL-1β cytokine. Although it might be expected that that PM_W_ would also elicit inflammasome activation through such mechanisms, there was no accompanying increase in IL-1β secretion (Figure S9). This suggests that although IL1B expression was upregulated by PM_W_, this response alone may not necessarily result in inflammation in the intact lung.

Cytochrome P450, family 1, subfamily A, polypeptide 1 (CYP1A1), also known as aryl hydrocarbon hydroxylase (AHH), a member of the cytochrome P450 enzyme family, was also upregulated by PM_W_ (but not PM_B_) exposure, suggesting activation of xenobiotic metabolism pathways, which may either detoxify or activate PAHs to more bioactive forms.^61,62^ The observed upregulation of CYP1A1 is likely mediated by the aryl hydrocarbon receptor (AhR) in response to PAH exposure.^63^ It was previously reported that the hydrophobic and poorly soluble nature of PAHs, which are an abundant and diversified component of PM_W_, influences the expression of genes involved in the AhR signaling pathway.^63^ However, expression of the Aryl Hydrocarbon Receptor Repressor (AHRR), which is induced in a negative feedback mechanism to attenuate AhR activity,^64^ was not significantly dysregulated by exposure to PM_W_, suggesting that AhR activation by PM_W_ PAHs was not suppressed, allowing sustained upregulation of CYP1A1.

Other notable upregulated genes included nuclear receptor 4A3 (NR4A3), which codes for a p53 receptor that activates apoptosis,^65^ and triggering receptor expressed on myeloid cells-like 4 (TREML4), which encodes a macrophage receptor involved in regulation of innate immune responses.^66^

Finally, exposure to PM_W_ (but not PM_B_) caused downregulation of genes encoding members of the C-type lectin domain family (CLEC10A, CLEC12A, CLEC12B, CLEC1A, and CLEC7A), which function as pattern recognition receptors and play a role in innate immune responses.^67^ Likewise, PM_W_ exposure caused downregulation of CX3C motif chemokine receptor 1 (CX3CR1), which stimulates recruitment of immune cells in response to pathogens, and of major histocompatibility complex (MHC) proteins HLA-DPA1 (MHC complex class II, DP alpha 1) and HLA-DRA (HLA class II histocompatibility antigen), which are involved in antigen presentation. Exposure to PM_W_ (but not PM_B_) also downregulated Interleukin 33 (IL33), classified as an “alarmin” cytokine, which can activate immune and allergic responses,^68^ as well as Toll-like receptor 7 (TLR7) and Toll-like receptor 8 (TLR8), which play critical roles in antiviral immune responses.^69^ Downregulation of these cytokines and C-type lectins could impair innate immune responses in the lung.

Exposure to PM_W_ did not significantly affect expression of genes coding phagocytic receptors, including those coding for Fcγ receptors (FCGR1A, FCGR2A, and FCGR3A) and for the scavenger receptors CD36 and MARCO. Since exposure to PMW appeared to decrease phagocytosis of PS beads primarily by impairing binding (Figure 5G–I), this suggests that the effect was likely due to interactions of PM_W_ with phagocytic receptors, resulting in either blockade of binding sites or conformational changes that impaired binding of beads. Further studies are needed to assess and characterize the effects of interactions of model PM_W_ particles with phagocytic receptors.

In conjunction with the reduction in viability (Figure 4B) and mitochondrial membrane potential (Figure 4D), and the impairment of phagocytosis (Figure 5), all unique to, or more substantial after exposure to PM_W_ than to PM_B_, the findings of this study strongly suggest that exposure to WUI fire PM_0.1_ has considerably more negative impacts on lung macrophage health and function, and thus causes more significant adverse health effects, than exposure to biomass wildfire PM_0.1_. The most likely cause of the greater toxicity of WUI fire PM is its chemical composition, particularly the presence of highly toxic alkyl and oxygenated PAHs. This and the much greater carcinogenic potential (BaP_Eq_) of PM_W_ (483 ng/m^3^) compared to PM_B_ (24 ng/m^3^) (Figure S6) indicate that PM_W_ may thus represent a more serious public health hazard than PM_B_. The public health implications of the greater chemical complexity and toxicity of WUI fire PM relative to biomass fire PM are concerning, particularly given the rapid encroachment of urban development into wildland areas, combined with increasing global temperatures and drought conditions. A comprehensive assessment of real-world WUI fire PM physicochemical properties and the potential health impacts of WUI fire PM exposure is urgently needed to provide risk assessors and regulators with the data needed to evaluate the potential health risks associated with WUI fires.

## Supplementary Material



## Notes

Supplementary methods; total elemental composition of the PM_0.1_ fractions of PMB and PMW (Figure S1); PAH-type profiles of the PM_0.1_ fractions of PM_B_, PM_W_, and PM_HDPE_ (Figure S2); particle number concentration as a function of time and temperature (5.52 nm–209.1 nm) (Figure S3); particle number concentration as a function of time and temperature (523 nm–19800 nm) (Figure S4); aerosol mass-size distribution and concentration for all size fractions of PW and PW+HDPE (Figure S5); total Volatile Organic Compounds (VOCs) as a function of time and temperature (Figure S6); carbon monoxide concentration as a function of time and temperature (Figure S7); PAH BaP_Eq_ profiles of the PM_0.1_ fractions of PM_B_, PM_W_, and PM_HDPE_ (Figure S8); cytokines release by THP-1 macrophages after exposure to PM_B_, PM_W_, or controls (Figure S9); colloidal characterization of PMB and PMW (Table S1); summary of dosimetry and in vitro dose assessments (Table S2); detected PAH and OPAH members (Table S3) (PDF)
