# Supplementary material for: Wildland-Urban Interface Fires: Toxic Physicochemical Properties of Emitted Particulate Matter and Impacts on Lung Macrophages
Source: Environ Sci Technol. 2026 Apr 1;60(14):10562–75. doi: 10.1021/acs.est.5c16340 (PMC13085806; doi:10.1021/acs.est.5c16340)
Supplement: Supplementary file 1 [file es5c16340_si_001.pdf]

Supporting Information

Number of Pages: 27
Number of Figures: 9
Number of Tables: 3

Wildland-urban interface fires: toxic physicochemical properties of emitted particulate matter and impacts on lung macrophages

Glen M. DeLoid [a, †], Lila Bazina [a,b, †], Leonardo Calderon [a], Georgios A. Kelesidis [a,b,c], Jose Guillermo Cedeno Laurent [a,b], Irini Tsiodra [d], Nikolas Mihalopoulos [d,e], Luke Fritzky [f], Nachiket Vaze [a], Shuo Xiao [g], Audrey Gaskins [h], and Philip Demokritou [a,b]*

a. Nanoscience and Advanced Materials Center, Environmental and Occupational Health Sciences Institute (EOHSI), Rutgers University, Piscataway, NJ 08854, USA

b. Department of Environmental Occupational Health and Justice, School of Public Health, Rutgers University, Piscataway, NJ 08854, USA

c. Faculty of Aerospace Engineering, Delft University of Technology, Delft, the Netherlands

d. Institute for Environmental Research and Sustainable Development, National Observatory of Athens, Athens 15236, Greece

e. Environmental Chemical Processes Laboratory, Department of Chemistry, University of Crete, Heraklion 71003, Greece

f. New Jersey Medical School, Cancer Institute of New Jersey, Rutgers University, Newark, NJ 07103, USA

g. Department of Pharmacology and Toxicology, Ernest Mario School of Pharmacy, Rutgers University, Piscataway, NJ 08854, USA

h. Department of Epidemiology, Rollins School of Public Health, Emory University, Atlanta GA 30322

† These authors contributed equally to this work.

* Corresponding authors:
E-mail address: gd424@eohsi.rutgers.edu (G. DeLoid)
E-mail address: philip.demokritou@rutgers.edu (P. Demokritou)

Supplementary Methods

Generation, collection, size-fractionation, and extraction of simulated pure biomass wildfire $PM_{0.1}$ (PM_B) and WUI fire $PM_{0.1}$ (PM_W). The $PM_{0.1}$ (≤0.1 μm) size fractions of simulated pure biomass wildfire PM (PM_B) and WUI fire PM (PM_W) were synthesized using our WildFire Simulator (WiFS), previously described by the authors [1–5]. The WiFS platform allows investigation of the thermal decomposition behavior of materials under controlled combustion conditions and production and collection of the released particulate matter (PM) for physicochemical analysis and toxicological studies. The operational parameters for the combustion conditions were configured to simulate a flaming combustion scenario with the final temperature at 600 °C with a heating rate of 20 °C/min and ambient O_2 concentration (20.9 vol% in air). Starting weight of PW chips and PW chips + HDPE pellets (Poligroup, Bulgaria) was fixed at 100 mg in order to ensure reproducibility of the combustion atmosphere between replicate experiments. The $PM_{0.1}$ size fractions were collected on pre-cleaned 47 mm diameter polytetrafluoroethylene (PTFE) 2 μm pore filters (Pall Corporation, Port Washington, NY, USA) using the Harvard Compact Cascade Impactor (HCCI) [6]. The collected $PM_{0.1}$ were then extracted from the PTFE filters as previously described by Pal et al. [7]. Briefly, filters containing the collected $PM_{0.1}$ size fraction of PM produced by combustion of pinewood (biomass only wildfire $PM_{0.1}$, PM_B) or the mixture of pinewood and HDPE (representing WUI fire $PM_{0.1}$, PM_W) were placed in 50 mL beakers and immersed in 15 mL of 75% (v/v) ultrapure ethanol and subjected to bath sonication for 30-60 seconds.

Extracted PM_B and PM_W particle suspensions in 75% ethanol were washed three times with 75 mL of cell culture grade water (Cytiva, USA) by rotary evaporation to produce an ethanol-free aqueous suspension of PM_B and PM_W for toxicological studies. Efficiency for extraction of PM_B and PM_W, calculated via gravimetric analysis of

filters and dried suspensions, was ~99%. To create a background/vehicle control for toxicological studies, a sterile PTFE filter without PM was subjected to the extraction procedure described above.

Real time monitoring of WiFS emitted gases. Real time monitoring of WiFS emitted gasses during combustion of PW chips or PW chips + HDPE pellets, carbon monoxide (CO) emissions were monitored using a portable combustion analyzer (Bacharach PCA3, Pittsburgh, PA) and concentrations in ppm were recorded at 10 second intervals for the duration of the combustion process. Total Volatile Organic carbons (TVOCs) emitted during combustion were also monitored using a portable photo-ionization detector probe (TVOC probe, TG-502, GrayWolf Sensing Solutions, Shelton, CT). Concentrations of TVOCs were recorded as ppb and logged every 10 s throughout the duration of the combustion process.

Real time monitoring and physiochemical characterization of emitted PM. During combustion of PW chips or PW chips + HDPE pellets, PM emissions were monitored using a Scanning Mobility Particle Sizer (SMPS) Model 3080 (TSI Inc., Shoreview, MN) to measure particle number concentration of particles in the 5 to 300 nm range, and an Aerodynamic Particle Sizer (APS) Model 3321 (TSI Inc., Shoreview, MN) to measure number concentrations of particles ranging from 0.5 to 20 μm. To produce an aerosol with particle concentrations within measurement range of the SMPS, the emitted aerosol was diluted 200-fold with a Rotating Disk Thermodiluter Model 379020 A (TSI Inc., Shoreview, MN), as described previously by Sotiriou et al. (2015) [3]. SMPS measurements were taken every 2 minutes and APS measurements were taken every 10 seconds throughout the combustion process. Measured SMPS concentrations were adjusted to account for the 200-fold dilution factor.

Size-fractionated PM and from PW and PW+HDPE incineration ($PM_{0.1}$: aerodynamic diameter (d_{ae}) < 0.1 μm; $PM_{0.1\text{-}2.5}$: 0.1 μm ≤ d_{ae} < 2.5 μm; $PM_{2.5\text{-}10}$: 2.5 μm ≤ d_{ae} ≤ 10 μm , and PM_{10}: d_{ae} > 10 μm) collected using Harvard Compact Cascade Impactors (CCIs) were used to derive mass particle size distributions and concentrations as previously described [63]. Chemical analysis of the $PM_{0.1}$ fractions (PM_B and PM_W) included quantification of organic and elemental carbon (EC-OC) content using thermal-optical transmission, measurement of total

inorganic elemental composition by Inductively Coupled Plasma Mass Spectrometry (ICP-MS), and quantification of total and speciated polycyclic aromatic hydrocarbons (PAHs) using Gas Chromatography–Mass Spectrometry (GC/MS), as detailed in prior studies by the authors [89] and described below.

Organic and elemental carbon analysis of PM_B and PM_W from peak phase.

The analysis of organic and elemental carbon (EC-OC) was performed on PM_B and PM_W collected on quartz filters during the peak phase. One cm^2 punchout areas of the filters were created and analyzed using the thermal-optical transmission (TOT) technique with a Sunset carbon analyzer (Sunset Laboratory Inc., Portland, OR, USA) and the EUSAAR2 thermal protocol described in detail by Cavalli et al. [10].

Inorganic elemental analysis of PM_B and PM_W samples. The elemental particle composition at the individual particle level was determined by multi-element SP-ICP-TOF-MS (TOFWERK, Thun, Switzerland) as previously described [11–13]. Details are provided in supplementary materials.

Multi-element single particle composition analysis by SP-ICP-TOF-MS. Samples were introduced into the ICP with a 2DX autosampler (Element Scientific, Omaha, United States) and a MicroMist U-series Nebulizer (Thermo scientific, USA) connected via a Quartz Cyclonic Spray Chamber (Meinhard, USA) to the injector of the ICP torch. The instrument operating parameters and the monitored isotopes were previously published in Cedeño Laurent et al., 2024 [14].

Element specific instrument sensitivities were measured with a series of multielement solutions prepared from a mixed multi-element ICP certified reference standard (0, 1, 2, 5, and 10 μgL^{-1} multi-element standard, diluted in 1% HNO_3, BDH Chemicals, Radnor, PA, USA). The transport efficiency was calculated via the known size method using a certified 60-nm Au ENMs (NIST RM 8013 Au, Gaithersburg, MD, USA) and a series of ionic Au standards (BDH Chemicals, West Chester, PA, USA) [15]. A 4.5% H2/He gas mixture was used as collision gas

to eliminate/minimize interferences and was optimized for 56Fe+ and 28Si+ signals. All data processing – signal thresholding (Poisson algorithm [16]) and split event correction - was performed using Tofpilot (Version 2.11.3, TOFWERK, Thun, Switzerland). The mass and size detection limits assuming pure metal and metal oxide phases were previously published in Cedeño Laurent et al., 2024 [14]. All samples and UPW blanks were analyzed in triplicates and data was acquired for 200 s for each replicate. After verifying the reproducibility of the single particle elemental composition and number concentrations among the replicates, the three replicates were combined to achieve comprehensive analysis due to limited detection events of certain elements. Select elemental ratio distributions were determined on a particle-by-particle basis taking into account all particles.

Nanoparticle classification analytical methods. The detected NMs were classified into single- and multi-metals (smNMs and mmNMs). The mmNMs were further classified into clusters of mmNMs of similar elemental composition using two-stage (e.g., intra- and inter-sample) automated agglomerative hierarchical clustering analysis performed in MATLAB as described in elsewhere [12,13]. Intra-sample clustering was performed on all metal masses in each NM, using average correlation distance, to generate clusters that best account for variance in NM metallic composition in each sample. The generated clusters were grouped into major clusters using an optimal distance cutoff. A cluster representative was determined for each major cluster as the mean of metal mass in individual NMs within each cluster taking into account all elements that occurred in at least 5% of NMs within the cluster. The mean intra-sample cluster composition was determined as the mean of metal mass fraction in all NMs in the cluster and was compared across samples. Inter-sample clustering was performed on the major cluster representatives identified in the intra-sample clustering to group/cluster the similar NM major clusters identified in the different samples. Major/similar clusters were identified using an optimal cutoff. The optimal distance cutoff was determined by maximizing the mean silhouette score for each sample. The silhouette score is a measure of how similar a NM is to its own cluster (cohesion) compared to other clusters (separation) [17]. Thus, silhouette coefficient provides a measure of how well each NM has been classified [17]. The silhouette score ranges from −1 to +1, where a high value indicates that a NM is well matched to its own cluster and poorly matched to neighboring

clusters (far away from the neighboring clusters). In contrast, 0 indicates that a NM is on or very close to the decision boundary between two neighboring clusters. A negative value indicate that a NM might have been assigned to the wrong cluster. For the first stage hierarchical clustering, major clusters were determined for a range of distance cutoff from 0.3 to 0.9 with an increment of 0.05. Then, the mean silhouette coefficient was determined for each distance cut off. The optimal distance cutoff was selected as that resulting in the highest mean silhouette score. For the second stage hierarchical clustering, the distance cutoff values varied between 0.02 and 0.6 with an increment of 0.001. Select elemental ratios were determined on a particle-by-particle basis taking into account all particles containing the two elements, and the elemental ratio distribution was determined. The number concentration (NM g^{-1}) of the total, smNMs, mmNMs, and cluster members were determined according to SP-ICP-MS theory 5. Finally, heat maps were generated by comparing the number concentration of NMs in each major cluster among the different samples.

PAH and OPAH analysis of PM_B and PM_W from peak phase. The protocol described in Tsiodra et al. [18] was used to perform quantitative analysis of polyaromatic hydrocarbons (PAHs) and oxygenated PAHs (OPAHs) in the PM_B and PM_W fractions from the peak phase of combustion. The analysis focused on the identification of 31 PAHs and 7 OPAHs with molecular weight between 178 and 300 g mol^{-1} (**Table S3**). In brief, the aqueous suspension of size fractionated PM samples were spiked with a known amount of deuterated PAHs (16 members) mixture (CPA Chem). These members were used as surrogate standards for the identification of PAHs, for the calculation of the recovery efficiencies and were added prior to extraction. PAHs were extracted using pressurized liquid extraction with an accelerated solvent extractor system (ASE-300, Dionex) and solvent mixture 50:50 n-hexane -dichloromethane. The sample extracts were purified through a silica column and the selected fractions were collected using different polarity solvents. The fraction which contained PAHs was eluted using 11 mL of n-hexane/ethyl acetate (8:2 v/v) and was condensed to the reduced volume of 0.1 mL. At the end of the experiment a specific amount of $[^2H_{12}]$perylene was added as internal standard. During the day of the analysis, to quantify the desirable compounds a mixture of native and deuterated PAHs was injected into the gas chromatography/mass

spectrometry (GC/MS) instrument for the calculation of the relative response factors (RRF). The parent PAHs and OPAHs species are presented in **Table S3**. The analysis was performed in a gas chromatography/mass spectrometry (GC/MS) system (Agilent 7890 GC), using an HP-5MS capillary column (30 m × 0.25 mm i.d. × 0.25 μm phase film) coupled with an Agilent 5975C mass selective detector, operated in full scan mode. Details regarding the GC/MS analysis are provided in Tsiodra et al., 2025 [18]. Blank quartz filters used in the PM sampling were analyzed to determine background contamination.

Endotoxin and microbiological sterility testing of PM_B and PM_W samples. Endotoxin levels in PMB and PMW were assessed with the HEK-Blue™ LPS Detection Kit 2 (Invivogen, San Diego, CA, USA) according to the manufacturer's instructions as previously described in Lizonova et al., 2024 (20). HEK-Blue™-4 cells were grown in complete media containing Dulbecco's modified Eagle's High Glucose medium (DMEM, Life Technologies, Inc., Carlsbad, CA) without phenol red, supplemented with 10% heat-inactivated ultra-low endotoxin fetal bovine serum (FBSLE, Corning, Inc.), 2 mM L-alanyl-L-glutamine (Corning, Inc.), 100 U/mL penicillin and 100 µg/mL streptomycin (Life Technologies, Inc., Carlsbad, CA), 100 µg/mL Normocin, and the selection antibiotics provided in the kit. Twenty µL of each sample to be tested, including a 100 µg/mL suspension of PMB and PMW, a blank filter extraction control suspension (to assess endotoxin contamination from the CCI filter and rotavap extraction process), and endotoxin-free water alone (to assess background endotoxin), was pipetted into designated wells of a tissue culture-treated 96-well plate. Endotoxin standards ranging from 0.01 to 1 EU/mL were prepared using endotoxin from Escherichia coli serotype 055 and added to designated wells. A suspension of HEK-Blue™-4 cells (160 µL containing 48,000 cells) was then added to all wells, and the plate was incubated for 20 hours at 37°C and 5% CO2. Following incubation, 40 µL of supernatant from each well was transferred to a new detection plate, and 160 µL of Quanti-Blue (QB) reagent mixture was added. The plate was then incubated at 37°C until color development occurred (approximately 2-4 hours). Absorbance was recorded at 620 nm, and endotoxin concentrations were determined using a standard curve based on the

absorbance values from the endotoxin standard wells. Additionally, PMB and PMW samples spiked with an additional 0.1 EU/mL of endotoxin were analyzed to evaluate potential interference.

The microbiological sterility of PMB, PMW, and vehicle control were assessed according to the WHO standards specified in the international pharmacopoeia, as described in our prior investigations (21, 22). Samples were suspended at a concentration of 1 mg/mL, and 1 mL of each suspension was mixed with 10 mL of fluid thioglycolate medium at a pH of 6.9–7.3. The solutions were then incubated at 37 °C for 14 days and examined daily for indications of bacterial growth. Throughout the 14-day incubation period, each sample and control was systematically evaluated for bacterial and fungal colonies using the pour plate technique with potato dextrose agar (PDA) and plate count agar (PCA).

Preparation and colloidal characterization PM_B and PM_W suspensions. Preparation and colloidal characterization of PM_B and PM_W dispersions were carried out as previously described by the authors [19–21]. In brief, 1 mg/mL suspensions of PM_B and PM_W in HyClone HyPure Endotoxin-free water (Cytiva, USA) were subjected to one-minute rounds of cup-horn sonication (Branson Sonifier S–450D, 400 W, with a 3-in. cup horn, and a power output of 1.26 W), followed by 30 seconds of vortexing. At the end of each round, the hydrodynamic diameter (z-average, d_H) of the suspension was measured using dynamic light scattering (DLS, Malvern Zetasizer Nano ZS, Malvern Panalytical Inc., Westborough, MA), and additional rounds were performed until the decrease in d_H between rounds was less than 5%. The total sonication time to reach that point and the power output of the sonicator were used to calculate the critical delivered sonication energy (DSEcr in J/mL) for the PM_B and PM_W suspensions. The DSEcr is thus the total energy (J/mL) required to produce a stable aqueous dispersion of particles with the smallest possible d_H (i.e., minimum possible agglomeration). Subsequently, 1 mg/mL aqueous dispersions of PM_B and PM_W were sonicated to the DSEcr, followed by dilution in cell culture media (RPMI+10% FBS) to produce the final treatment suspensions at the desired PM_B and PM_W concentrations for toxicological studies, determined as described below.

Pulmonary deposition modeling of PM_B and PM_W using the Multiple-Path Particle Dosimetry (MPPD) model. The Multiple Path Particle Dosimetry (MPPD) model (V3.04) was used to calculate the rate of mass deposition of PM_B and PM_W per unit surface area (i.e., μg/cm²/min) in the pulmonary region of the lung in an average human as a function of exposure time at an ambient concentration of 375 μg/m³. The selection of this ambient $PM_{0.1}$ concentration was based on and comparable to ambient $PM_{2.5}$ levels during the June 2023 Canadian wildfire event in the New Jersey/New York area, which were measured by our lab with a peak of 317 μg/m³ [14], and reported at 400 μg/m³ in Syracuse, NY [22], as well as $PM_{2.5}$ measured during the 2018 Camp fire in California (263 μg/m3) [23], and during the 2020 California wildfires, which reached 500 μg/m³ [14], and the fact that the $PM_{0.1}$ fraction typically makes up the majority of wildfire $PM_{2.5}$. The MPPD analysis was conducted using the methodology and parameters outlined by Lizonova et al., with a functional residual capacity of 3300 mL, tidal volume of 625 mL, head volume of 50 mL, nasal respiratory rate of 12 breaths per minute, and inspiratory fraction of 0.5 [24] . The aerosol effective density (ρ_{eff}) of PM_B and PM_W employed in the MPPD model was 1.1 g/cm³, which was the ρ_{eff} reported for organic carbon PM by Ouf et al. [25]. The mass mean aerodynamic diameter (MMAD) employed was 0.5 μm. The mass deposition rates (μg/cm²/min) for PM_B and PM_W calculated using the MPPD model were then used to calculate total mass deposition in the lung (μg/cm2) for exposure times of 0.5, 5, and 50 days, by multiplying the deposition rates by each duration (in minutes).

Calculation of administered PM_B and PM_W in vitro doses to match MPPD deposition doses using the Distorted Grid (DG) dosimetry model. We employed the distorted grid (DG) in vitro dosimetry model, previously developed by our lab [26], to determine administered concentrations of PM_B and PM_W in cell culture media that would result in 24 h mass depositions (μg/cm²) (i.e., doses delivered to cells) corresponding to pulmonary mass depositions that would occur in the human lung after 0.5, 5, and 50 day exposures at ambient PM_B and PM_W of 375 μg/m³, calculated using the MPPD model as described above. The DG model simulates sedimentation and diffusion of particles in suspension based on particle size distribution and effective density

(ρ_{EV}), media density and viscosity, and the media column height in the cell culture well to determine the mass deposition of particles (µg/cm^2) (i.e., the dose delivered to cells), at a given initial particle concentration (µg/cm^3) (i.e., the administered dose) as a function of time, as previously described in detail [20,26]. The PM_B and PM_W size distributions were determined by DLS, as described above. The ρ_{EV} employed for PM_B (biomass fire $PM_{0.1}$) was 1.565, previously determined for WFPM $PM_{0.1}$ collected during the 2023 Canadian wildfire event by Bazina et al., 2025 [27]. The $\boldsymbol{\rho}_{EV}$ for PM_W (WUI fire $PM_{0.1}$) was estimated as the average of the $\boldsymbol{\rho}_{EV}$ of PM_B and the $\boldsymbol{\rho}_{EV}$ of PM produced by incineration of HDPE (plastic) alone (0.96 g/cm^3), reported by Watson et al., 2017 [2]. The density and viscosity of RPMI media with supplements employed in the DG model were those previously measured and reported by the authors [20,26]. To determine administered concentrations required to match the desired deposited doses calculated using the MPPD, the DG model was first run for suspensions of PM_B and PM_W at an arbitrary administered concentration of 100 µg/mL. The 24 h deposition (µg/cm^2) calculated by the DG model at 100 µg/mL (Dep_{DG100}) was then used to calculate the actual administered concentrations required to achieve the target deposited doses (Dep_{Target}) using the following equation:

$$C = 100 \times (Dep_{Target}/Dep_{DG100})$$

Culture and preparation of THP-1 macrophages. Human THP-1 monocytes, acquired from ATCC (Manassas, VA), were cultured in RPMI medium supplemented with 1% Amphotericin B, 100 U/mL penicillin, 100 µg/mL streptomycin (Life Technologies, Inc., Carlsbad, CA), 10% heat-inactivated fetal bovine serum (FBS), and 10 mM HEPES (Corning, Inc.). THP-1 monocytes were differentiated to human macrophages with phorbol 12-myristate-13-acetate (PMA) (Life Technologies, Inc., Carlsbad, CA) following the method developed by Daigneault et al.[28], which was shown to produce macrophages that closely resemble primary human macrophages in morphology, phenotypic markers, and functionality. Briefly, THP-1 monocytes were dispersed at a concentration 4.0×10^5/mL in RPMI + 10% FBS containing 100 nM PMA. The cells were subsequently dispensed into 96-well black-walled imaging plates (BD, Franklin Lakes, NJ) at a density of 8.0×10^5 cells per well and

plates were incubated at 37˚C, 5% CO_2 for three days. Cells were then rinsed with Phosphate Buffered Saline (PBS) (Corning, Inc.) and incubated for 4 day resting period in RPMI + 10% FBS without PMA.

Exposure of THP-1 macrophages to PM_B and PM_W. Suspensions of PM_B and PM_W in RPMI media (with supplements as detailed above) were prepared at concentrations that would result in 24 h mass depositions (μg/cm^2) (i.e., doses delivered to cells) corresponding to pulmonary mass depositions that would occur in the human lung after 0.5, 5, and 50 day exposures at ambient PM_B and PM_W of 375 μg/m^3, which were calculated using the MPPD and DG models, as described above. Culture media was aspirated from mature THP-1 macrophages prepared in 96-well plates, cells were washed once with 200 μL PBS, and 200 μL of either PM_B or PM_W suspension at each concentration, vehicle control, or fresh media was dispensed in each well. Plates were then incubated for either 4 h (for oxidative stress assessment, see below) or 24 h (all other assessments) at 37˚C with 5% CO_2.

Evaluation of cell membrane integrity (LDH release). Cellular membrane integrity was assessed by measuring lactate dehydrogenase (LDH) levels released to the culture medium, using the CyQUANT LDH Cytotoxicity Assay (Thermo Fisher, Waltham, MA) according to the manufacturer's instructions. Briefly, LDH substrate provide in the kit was dissolved in 11.4 mL of provided ultrapure water and added to 0.6 mL provided assay buffer to prepare the assay reaction mixture. Following 24-hour exposure of adherent PMA differentiated macrophages to PM_B, PM_W, media alone (untreated – Spontaneous LDH release), or 45 min incubation with provided lysis buffer (positive control – Maximum LDH release), cell media from wells was collected in 1.5 mL tubes, and centrifuged at 3000 × g for 5 minutes to pellet cell debris. Fifty μL of the supernatant from each tube was dispensed in triplicate wells of a new 96-well plate along with 50 μL of the reaction mixture. Plates were then incubated at room temperature for 30 minutes before 50 mL of stop solution was added to each well to end the reaction. Absorbance was measured at 490 nm (A490) and 680 nm (A680) using a SpectraMax M-5 reader and SoftMax Pro software (Molecular Devices). A680 values were subtracted from A490 values to correct for

instrument background. Percent cytotoxicity was calculated by subtracting background-corrected spontaneous LDH release values from background-corrected treatment values, dividing by total LDH activity (Maximum LDH release – Spontaneous LDH release), and multiplying by 100. LDH activity in culture media without particles and with particles at the maximum dosage were also measured to evaluate possible interference caused by the particles [19].

Evaluation of cell viability (mitochondrial metabolic enzyme activity). Following 24 h exposures of PMA differentiated macrophages to PM_B, PM_W, or media only (untreated - 100% viability control), cells were rinsed with 200 μL/well PBS and incubated at room temperature with 100 μL/well PrestoBlue® reagent (Thermo Fisher) for 30 minutes. Fluorescence intensity was quantified at 570nm excitation/610 nm emission with a SpectraMax M-5 microplate reader and SoftMax Pro software (Molecular Devices). Cell viability was calculated as the percentage of the fluorescence signal in untreated (100% viable) cells. To evaluate possible interference with assay measurements from PM_B or PM_W particles, culture medium without particles and with particles at the maximum dose were also analyzed.

Evaluation of oxidative stress (reactive oxygen species production). Intracellular reactive oxygen species (ROS) generation was quantified using the CellROX® Green assay (Thermo Fisher, Waltham MA) according to the manufacturer's instructions. Briefly, adherent PMA-differentiated macrophages in 96 well plates were exposed to PM_B, PM_W, or vehicle for 4 hours, or to 1.25 mM menadione (positive control) for 2 hours, at 37 °C, 5% CO2. The cells were then washed with 200 μL/well PBS and incubated with 100 μL/well of the assay reaction mixture at 37 °C, 5% CO2 for 30 minutes. The reaction mixture was then replaced with 200 μL/well PBS and fluorescence was measured at excitation/emission wavelengths 480 nm/520 nm. ROS levels in the treated cells were expressed as fold change in fluorescence relative to untreated controls.

Evaluation of mitochondrial membrane potential. Mitochondrial membrane potential in adherent PMA-differentiated THP-1 macrophages was assessed using the JC-1 Mitochondrial Membrane Potential Detection Kit (Biotium, USA) following the manufacturer's protocol. A positive control was generated by incubating THP-1 macrophages in designated wells with 50 µM carbonyl cyanide m-chlorophenylhydrazone (CCCP) (Millipore Sigma, Burlington, MA) diluted in RPMI for 5 minutes at 37°C and 5% CO_2. Following treatments/exposures cells were washed with 200 µl PBS, the provided kit reagents were added to wells, and the plate was incubated at 37 °C for 15 minutes. A SpectraMax M-5 microplate reader with SoftMax Pro collection and analysis software (Molecular Devices) was used to measure fluorescence at 485 ex/435 em (green) and 550 ex/699 em (red). Red and green fluorescence in culture medium with and without PM_B or PM_W particles at the highest doses were measured to assess potential effects of the particles on the fluorescence signals. Background red and green fluorescence (in empty wells) were subtracted from test well red and green fluorescence values, respectively, and the ratio of red fluorescence (mitochondrial JC-1 aggregates, healthy cells) to green fluorescence (cytoplasmic JC-1 monomer), and indicator of mitochondrial membrane potential, was calculated for each well.

Assessment of cytokine/chemokine release. After 24 hour exposure of cells to PM_B, PM_W, vehicle, or 100 ng/mL LPS (positive control) , cell culture supernatants were collected and shipped on dry ice for assessment of cytokines/chemokines utilizing the Human Cytokine Array / Chemokine Array 48-Plex (HD48A) assay (Eve Technologies, Calgary, AB). The cell culture supernatants were prepared following instructions provided by Eve Technologies. In summary, supernatants were collected in 1.5 mL microcentrifuge tubes and subjected to centrifugation at 3000 × g for 10 minutes to remove cell debris. Supernatants were then transferred to new 1.5 mL tubes and stored at -80 °C until shipment. The HD48 assay allows the measurement of 48 cytokines and chemokines, including fibroblast growth factor 2 (FGF-2), interleukin-1alpha (IL-1A), monocyte chemotactic protein 1 1 (MCP-1), macrophage-derived chemokine (MDC), and tumor necrosis factor-alpha (TNF- α).

Assessment of innate immune function in THP-1 macrophages. Suspensions of unopsonized bead suspensions were prepared as previously described [29,30]. Briefly, 100 µL of stock suspension (20 mg/mL) of 1.0 µm biotin-labeled green fluorescent FluoSpheres™ Microspheres (Thermo Fisher, Waltham MA) was diluted 1/10 in PBS in a 1.5 mL Eppendorf tube and centrifuged for 5 minutes at 5,000 × g. The supernatant was aspirated (to remove sodium azide added by the manufacturer as preservative) and the pellet was resuspended in 0.5 mL of PBS. The suspension was then subjected to bath sonication for 15 minutes and diluted further in PBS to a final concentration of $4x10^8$/mL in complete RPMI1640 (without FBS) and 0.3% BSA (Millipore Sigma, Burlington, MA). The suspension was then subjected to shaking at 100 rpm and incubated for 15 minutes at 37°C to allow binding of BSA. A 0.5 µL stock of 15 µM cytochalasin D (Cyto-D) (Millipore Sigma ,Burlington, MA) was added to 0.5 mL of the final unopsonized bead solution to create a positive control unopsonized bead suspension.

The authors' previously established techniques [29,30] were followed to conduct the phagocytosis assay. In summary, adhered PMA differentiated macrophages were suspended in 10 µg mL−1 of HCS CellMask™Blue dye diluted in complete RPMI1640 (without FBS) (Life Technologies in Carlsbad, CA) and incubated at 37°C, 5% CO_2 for 40 minutes. After incubation, the reagent was discarded and cells were treated with PM_B, PM_W, or vehicle and incubated for 24 hours. Positive controls were treated with 15 µM Cytochalasin D for 30 minutes.

At the end of treatments/exposures, cells were washed with 200 µL of PBS and 50 µl/well of opsonized bead suspension (with or without Cytochalasin D) was added to wells. Macrophages were incubated with bead suspension at 37°C, 5% CO2 for 40 minutes to allow binding and internalization. Cells were then rinsed with cold PBS and incubated at 4 °C for 30 minutes with 100 µL/well of 5 µg/mL AlexaFluor568-streptavidin (Life Technologies, Inc., Carlsbad, CA) in PBS with 1% BSA. Cells were then washed with cold PBS and fixed with 4% formaldehyde for 10 minutes. Subsequently, the cells were rinsed with PBS and incubated at room temperature for 60 minutes with 100 µL/well of 2 µg/mL Hoechst 33342 nuclear dye (Life Technologies, Inc.,

Carlsbad, CA) in PBS. Prior to confocal imaging, the cells were washed once with PBS and stored with 200 μL/well of fresh PBS at 4 °C.

Confocal images were captured using the SP8 LIGHTNING confocal microscope (Leica Microsystems, USA), and custom MATLAB software (The MathWorks, Inc., Natick, MA, USA) was employed to process the collapsed confocal stack images for cell analysis. Image analysis followed previously established methodologies [29,30]. The MATLAB software segmented Hoechst/CellTracker Blue images to identify cells, counted the number of beads per cell using the green emission channel, and assessed bead localization relative to cell boundaries via the red emission channel. Pre-processing steps for Hoechst/CellTracker Blue images included noise reduction, contrast enhancement, and correction for uneven field brightness. A gradient-assisted watershed segmentation algorithm was utilized to identify and label individual cells. The green and red fluorescent bead images underwent sharpening and background noise removal prior to watershed segmentation, which identified and labeled individual particle objects. Particles were classified as "external" if they overlapped with objects in the red channel; and were otherwise labeled as "internal." Particle-cell associations were determined by identifying objects that shared pixels with cell objects. Total beads (bound + internal)/cell, internal beads/cell, and % internalization were calculated for each PM_B, PM_W, and control well.

Expression analysis by RNA-seq. Following 24 h exposures of PMA differentiated macrophages to PM_B, PM_W, or media only, lysates were collected. Briefly, cells were washed with 200 μl of cold PBS per well. The PBS was then replaced with 33 μl of TRIzol (Thermo Fisher, Waltham, MA) per well. The lysates were then mixed to ensure homogenization before being collected. RNA was extracted and purified from lysates by Invitrogen PureLink RNA Mini Kit (Thermo Fisher, Waltham, MA) according to manufacturer's instructions. RNA quality was assessed using a Nanodrop spectrophotometer (Thermo Fisher Scientific, USA). All samples met the required quantity and quality parameters and were shipped to Novogene (Durham, NC) for RNA-seq analysis. PolyA-enriched mRNA sequencing by Novogene was performed to capture and analyze the transcriptome. Additionally,

for each experiment, 20 to 30 million paired-end reads were generated and sequencing reads were mapped to the human reference genome (GRCh38.p14) [31].

Supplementary Figures

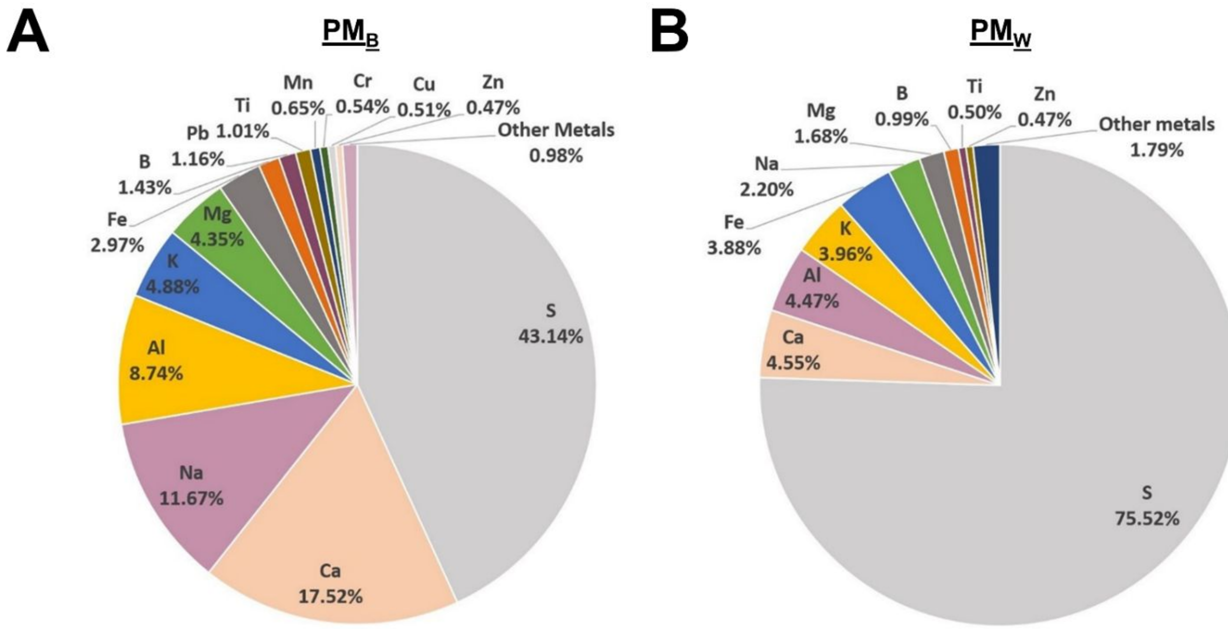

Figure S1. Total elemental composition of the $PM_{0.1}$ fractions of PM_B and PM_W.

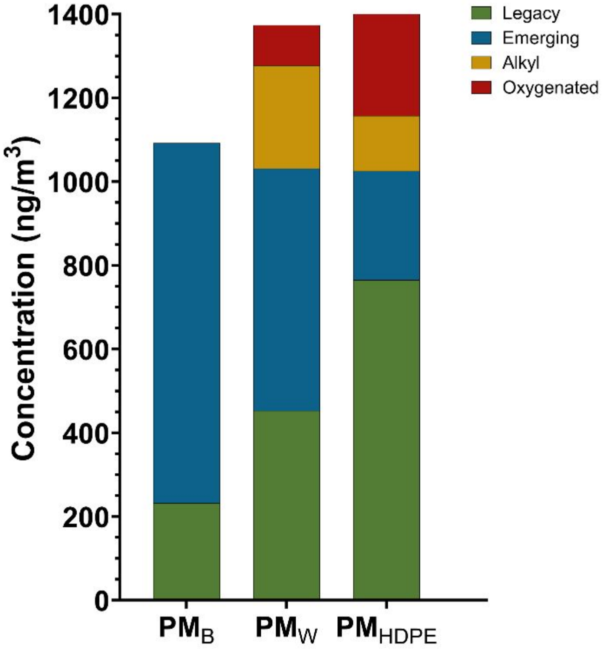

Figure S2. PAH type profiles of the $PM_{0.1}$ fractions of PM_B, PM_W, and PM_{HDPE}. The PM_{HDPE} PAH profile is reproduced here from Das et al. [32].

.

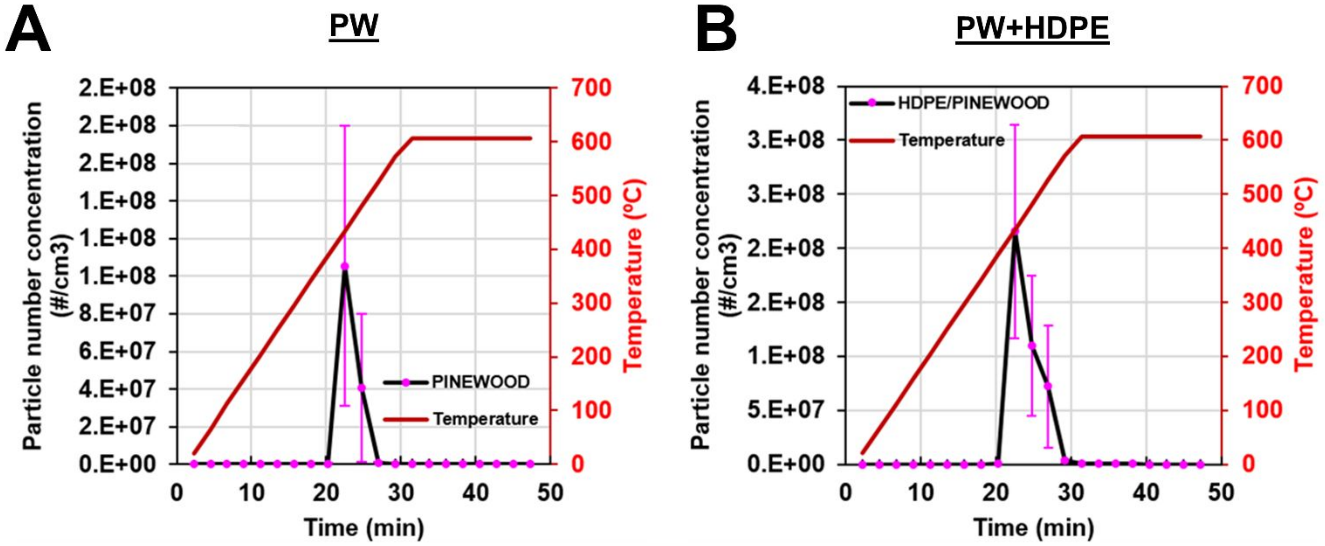

Figure S3. Particle number concentration as a function of time and temperature (5.52 nm – 209.1 nm) from SMPS

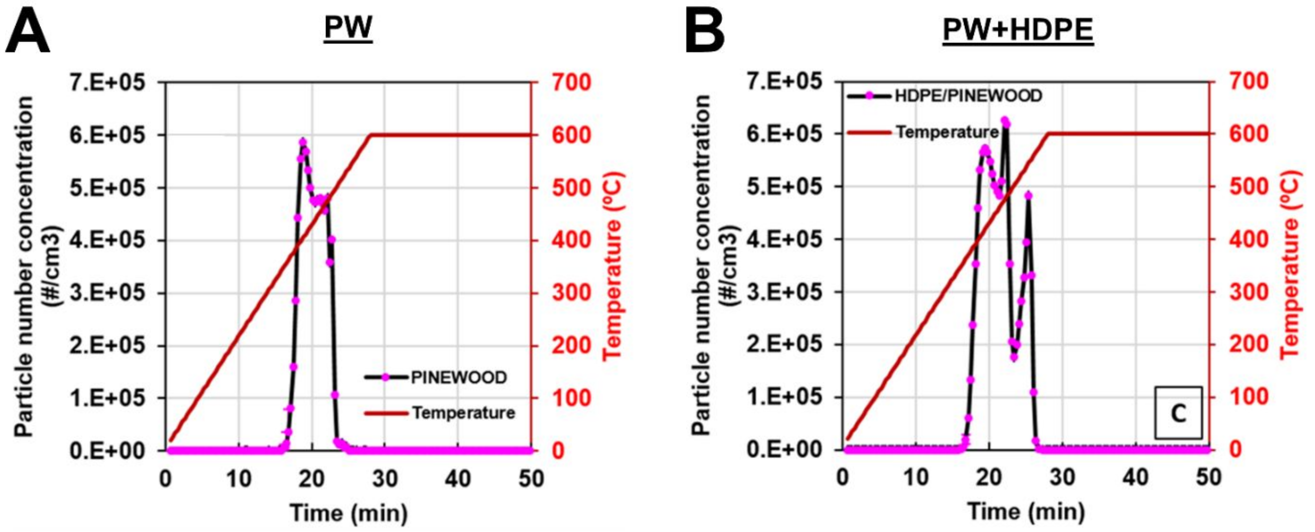

Figure S4. Particle number concentration as a function of time and temperature (523 nm – 19800 nm) by APS.

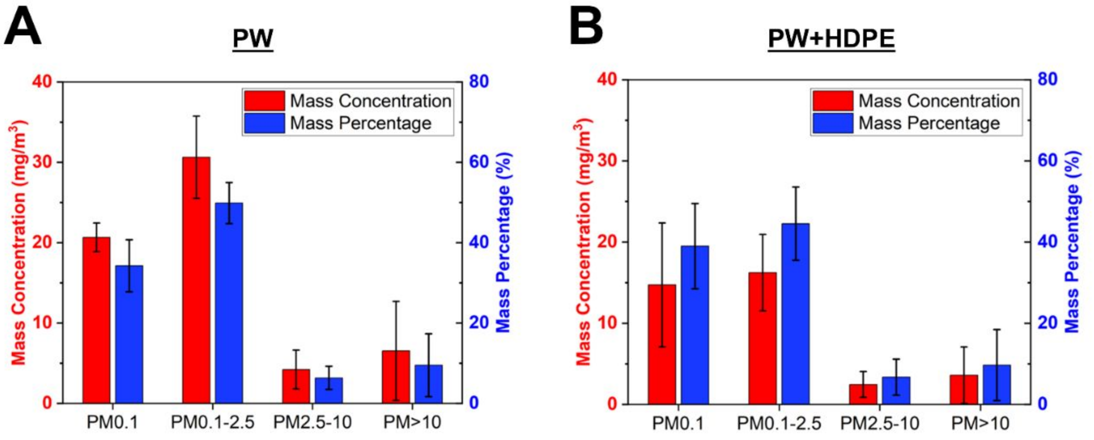

Figure S5. Aerosol mass-size distribution and concentration for all size fractions of PW and PW+HDPE

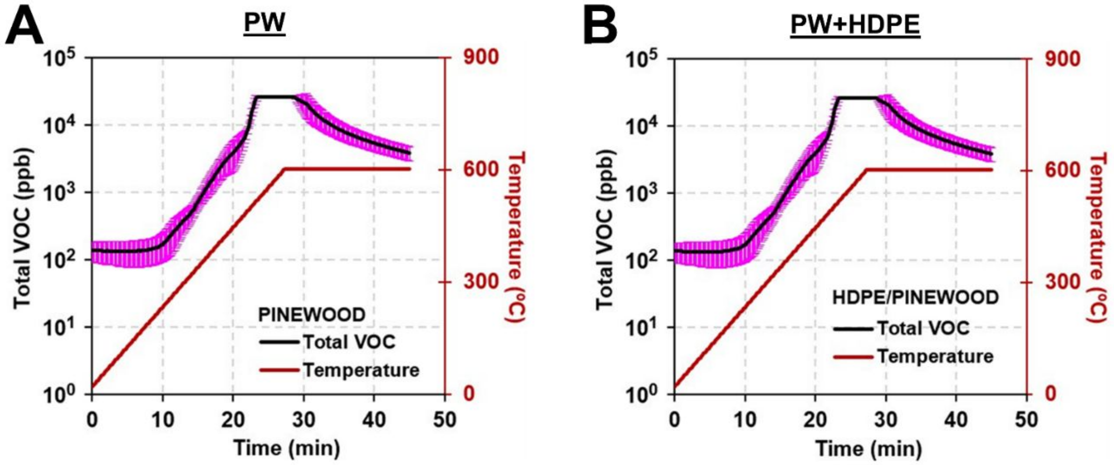

Figure S6. Total Volatile Organic Compounds (VOCs) as a function of time and temperature.

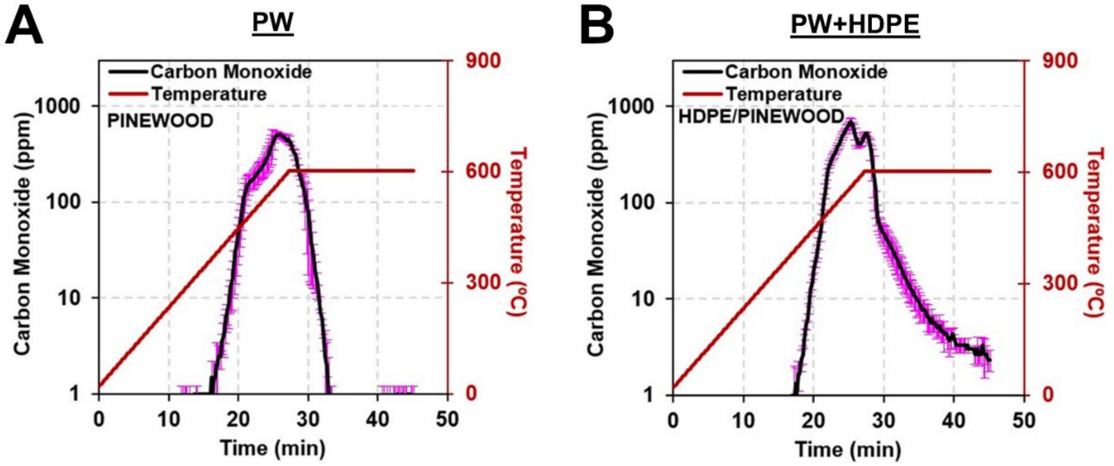

Figure S7. Carbon monoxide concentration as a function of time and temperature.

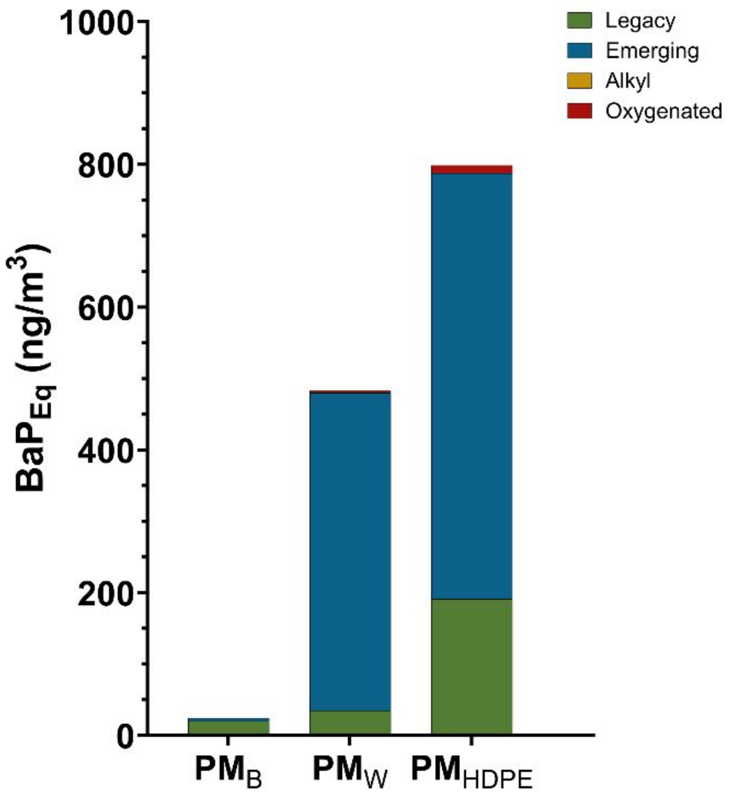

Figure S8. PAH BaP_{Eq} profiles of the $PM_{0.1}$ fractions of PM_B, PM_W, and PM_{HDPE}.

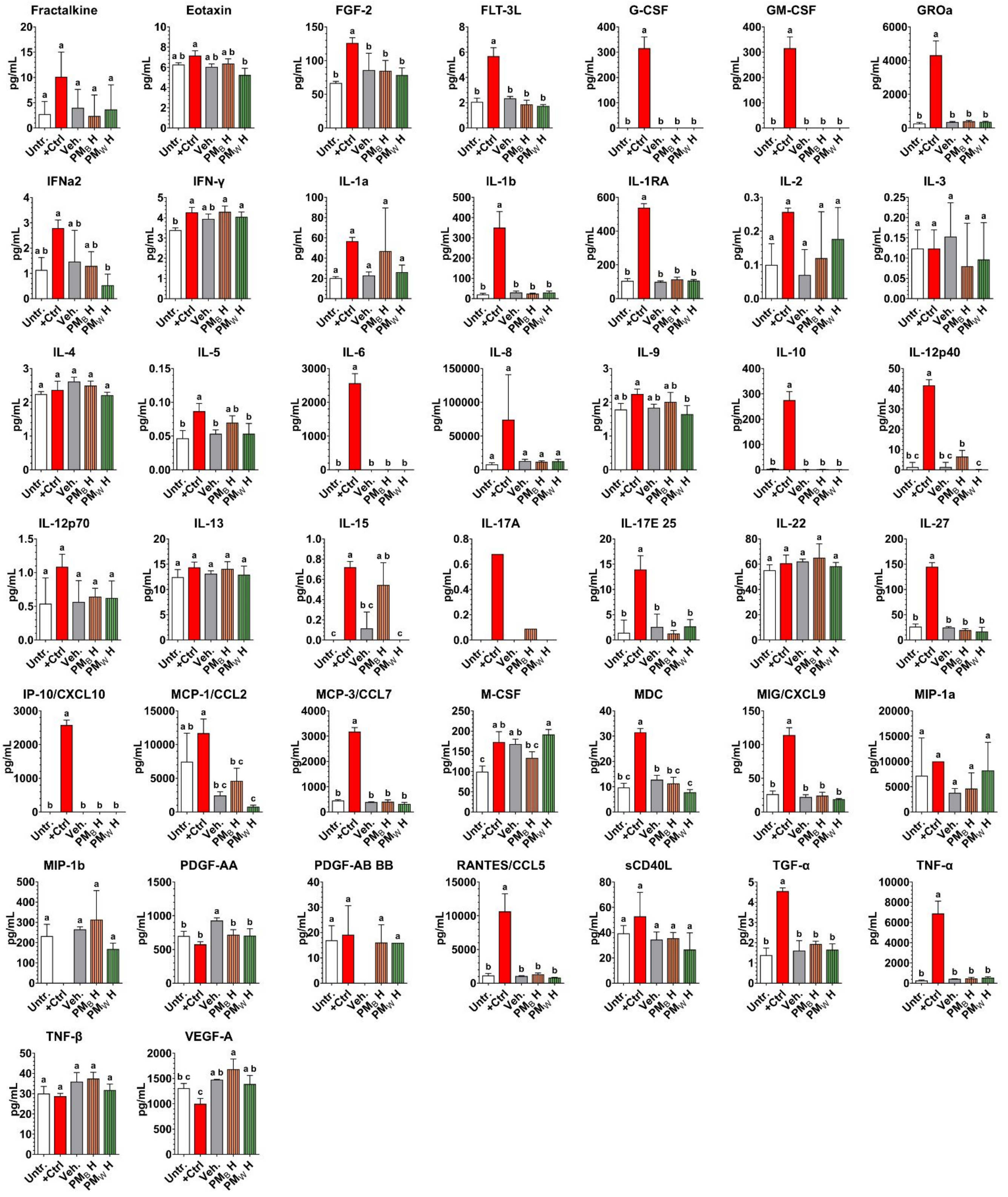

Figure S9. Cytokines release by THP-1 macrophages after exposure to PM_B, PM_W, or controls

Supplementary Tables

Table S1. Colloidal characterization of PM_B and PM_W in water and cell culture media at DSE_{cr}. d_H: hydrodynamic diameter, PdI: polydispersity index, ζ: zeta potential, σ: specific conductance (σ), ρ_{EV}: effective density in suspension.

Particle	Media	Time (h)	Intensity weighted d_H (nm)	PdI	ζ (mV)	σ (mS/cm)	ρ_{EV} (g/cm^3)
**PM_B**	DI Water	0	421.9±30.5	0.199±0.020	-34.30±0.95	0.050±0.000	
	RPMI Media +10% FBS	0	59.0±50.5	0.358±0.002	-9.86±0.71	13.200±1.010	1.565*
		24	28.7±0.9	0.845±0.030			
**PM_W**	DI Water	0	238.5±1.2	0.318±0.029	-27.40±11.80	0.072±0.001	
	RPMI Media +10% FBS	0	31.6±12.4	0.561±0.233	-10.00±0.96	12.800±0.660	1.263**
		24	21.4± 0.4	0.409±0.004			

*The colloidal effective density (ρ_{EV}) of PM_B was taken from value previously determined by Bazina et al., 2025 [27].

**The ρ_{EV} of PM_W was estimated as the average of the ρ_{EV} of PM_B and the ρ_{EV} of PM produced by incineration of HDPE (0.96 g/cm^3), reported in Watson et al., 2017 [2].

Table S2. Summary of dosimetry and *in vitro* dose assessments: MPPD deposition rates and cumulative depositions in the lung over 0.5, 5, and 50 d at 375 µg/m^3, and corresponding *in vitro* administered doses of PM_B and PM_W PM_{01} employed in THP-1 toxicity and function studies.

	MPPD Deposition Rate (µg/cm^2/min)	Exposure Duration (d)	Target Delivered Dose from MPPD (µg/cm^2)	Deposited Fraction from DG model	Administered Concentration Matching MPPD delivered dose (µg/mL)
**PM_B ($PM_{0.1}$)**	2.75 x10^{-6}	0.5	1.98 x 10^{-3}	0.348	0.118
		5	1.98 x 10^{-2}		1.180
		50	1.98 x 10^{-1}		11.80
**PM_W ($PM_{0.1}$)**		0.5	1.98 x 10^{-3}	0.181	1.394
		5	1.98 x 10^{-2}		13.94
		50	1.98 x 10^{-1}		139.4

Table S3. Detected PAH and OPAH members.

Molecular Weight (g/mole)	PAHs	Abbreviation	TEF	IARC group*
178	Phenanthrene	Phe	0.001	3
178	Anthracene	Ant	0.01	3
192	Methyl-phenanthrenes (5 species, with TEF)	Σ-C1-Phe	0.002	
202	Fluoranthene	Flt	0.08	3
202	Pyrene	Pyr	0.001	3
216	11H-Benzo[a]fluorene	11BaFL	0.001	
216	11H-Benzo[b]fluorene/ 7H-Benzo[c]fluorene	11BbFL/7BcFL	10**	
216	Methyl-202 (3 species, with TEF)	Σ-C1-202	0.001	
234	Retene	Ret	0.005	
226	Benzo[ghi]fluoranthene	BghiF	0.01	
228	Benzo(a)anthracene	BaA	0.2	2B
228	Chrysene	Chr	0.1	2B
252	Benzo(b+j)fluoranthene	BbjF	0.8	2B
252	Benzo(k)fluoranthene	BkF	0.2	2B
252	Benzo(a)fluoranthene	BaF		
252	Benzo(e)pyrene	BeP	0.01	3
252	Benzo(a)pyrene	BaP	1	1
252	Perylene	Per	0.001	3
276	Indeno(123cd)pyrene	IP	0.1	2B
276	Anthanthrene	Anth	0.1	3
278	Dibenzo(ah)anthracene	DBahA	10	2A
276	Benzo(ghi)perylene	BghiP	0.009	3
300	Coronene	Cor	0.001	3
**Molecular Weight (g/mole)**	**OPAHs**	**Abbreviation**	**TEF**	**IARC group***

196	Xanthone	XAN	0.006	
208	9,10-Anthraquinone	(9,10)O_2ANT	0.018	2B
230	11H-benzo(a)fluorenone	11OBaFL		
230	6H-Benzo(de)anthracene-6-one	6OBdeA	0.004	
230	11H-benzo(b)fluoren-11-one	11OBbFL		
230	7H-Benzo(de)anthracen-7-one (Benzanthrone)	BAN	0.004	
254	6H-Benzo[cd]pyren-6-one	6OBcdP	0.32	

*Group 1: Carcinogenic to humans, Group 2A: Probably carcinogenic to humans, Group 2B: Possibly carcinogenic to humans, Group 3: Not classifiable as to its carcinogenicity to humans.

** TEF assuming that half of the combined 11BbFL/7BcFL peak (which cannot be separated) is 7BcFL, which has a TEF of 20, while 11BbFL has a negligible TEF.

*** mixture of deuterated internal standards: ([$^2H_8$] naphthalene, [$^2H_8$]acenaphthylene, [$^2H_{10}$]acenaphthene, [$^2H_{10}$]fluorene, [$^2H_{10}$]phenanthrene, [$^2H_{10}$]anthracene, [$^2H_{10}$]fluoranthene, [$^2H_{10}$]pyrene, [$^2H_{12}$]benzo[a]anthracene, [$^2H_{12}$]chrysene, [$^2H_{12}$]benzo[b]fluoranthene, [$^2H_{12}$]benzo[k]fluoranthene, [$^2H_{12}$]benzo[a]pyrene, [$^2H_{14}$]dibenzo[ah]anthracene, [$^2H_{12}$]benzo[ghi]perylene, [$^2H_{12}$]indeno[1,2,3-cd]pyrene).

References

(1) Singh, D.; Tassew, D. D.; Nelson, J.; Chalbot, M.-C. G.; Kavouras, I. G.; Demokritou, P.; Tesfaigzi, Y. Development of an Integrated Platform to Assess the Physicochemical and Toxicological Properties of Wood Combustion Particulate Matter. *Chem. Res. Toxicol.* **2022**, *35* (9), 1541—1557. https://doi.org/10.1021/acs.chemrestox.2c00183.

(2) Watson-Wright, C.; Singh, D.; Demokritou, P. Toxicological Implications of Released Particulate Matter during Thermal Decomposition of Nano-Enabled Thermoplastics. *NanoImpact* **2017**, *5*, 29–40. https://doi.org/10.1016/J.IMPACT.2016.12.003.

(3) Sotiriou, G. A.; Singh, D.; Zhang, F.; Wohlleben, W.; Chalbot, M.-C. G.; Kavouras, I. G.; Demokritou, P. An Integrated Methodology for the Assessment of Environmental Health Implications during Thermal Decomposition of Nano-Enabled Products. *Environ. Sci. Nano* **2015**, *2* (3), 262–272. https://doi.org/10.1039/C4EN00210E.

(4) Singh, D.; Wohlleben, W.; De La Torre Roche, R.; White, J. C.; Demokritou, P. Thermal Decomposition/Incineration of Nano-Enabled Coatings and Effects of Nanofiller/Matrix Properties and

Operational Conditions on Byproduct Release Dynamics: Potential Environmental Health Implications. *NanoImpact* **2019**, *13*, 44–55. https://doi.org/10.1016/J.IMPACT.2018.12.003.
(5) Singh, D.; Marrocco, A.; Wohlleben, W.; Park, H. R.; Diwadkar, A. R.; Himes, B. E.; Lu, Q.; Christiani, D. C.; Demokritou, P. Release of Particulate Matter from Nano-Enabled Building Materials (NEBMs) across Their Lifecycle: Potential Occupational Health and Safety Implications. *J. Hazard. Mater.* **2022**, *422*. https://doi.org/10.1016/J.JHAZMAT.2021.126771.
(6) Demokritou, P.; Lee, S. J.; Ferguson, S. T.; Koutrakis, P. A Compact Multistage (Cascade) Impactor for the Characterization of Atmospheric' Aerosols. *J. Aerosol Sci.* **2004**, *35* (3), 281–299. https://doi.org/10.1016/j.jaerosci.2003.09.003.
(7) Pal, A. K.; Watson, C. Y.; Pirela, S. V.; Singh, D.; Chalbot, M. C. G.; Kavouras, I.; Demokritou, P. Linking Exposures of Particles Released From Nano-Enabled Products to Toxicology: An Integrated Methodology for Particle Sampling, Extraction, Dispersion, and Dosing. *Toxicol. Sci.* **2015**, *146* (2), 321–333. https://doi.org/10.1093/TOXSCI/KFV095.
(8) Singh, D.; Tassew, D. D.; Nelson, J.; Chalbot, M. C. G.; Kavouras, I. G.; Tesfaigzi, Y.; Demokritou, P. Physicochemical and Toxicological Properties of Wood Smoke Particulate Matter as a Function of Wood Species and Combustion Condition. *J. Hazard. Mater.* **2023**, *441*. https://doi.org/10.1016/J.JHAZMAT.2022.129874.
(9) José G. Cedeño Laurent; Hooman Parhizkar; Leonardo Calderon. Physicochemical Characterization of the Particulate Matter in New Jersey/New York City Area, Resulting from the Canadian Quebec Wildfires in June 2023. *Environmental Science & Technology*, 2024, (in review).
(10) Cavalli, F.; Viana, M.; Yttri, K. E.; Genberg, J.; Putaud, J. P. Toward a Standardised Thermal-Optical Protocol for Measuring Atmospheric Organic and Elemental Carbon: The EUSAAR Protocol. *Atmos. Meas. Tech.* **2010**, *3* (1), 79–89. https://doi.org/10.5194/AMT-3-79-2010.
(11) Alam, M.; Alshehri, T.; Wang, J.; Singerling, S. A.; Alpers, C. N.; Baalousha, M. Identification and Quantification of Cr, Cu, and As Incidental Nanomaterials Derived from CCA-Treated Wood in Wildland-Urban Interface Fire Ashes. *J. Hazard. Mater.* **2023**, *445*, 130608. https://doi.org/10.1016/J.JHAZMAT.2022.130608.
(12) Baalousha, M.; Wang, J.; Erfani, M.; Goharian, E. Elemental Fingerprints in Natural Nanomaterials Determined Using SP-ICP-TOF-MS and Clustering Analysis. *Sci. Total Environ.* **2021**, *792*, 148426. https://doi.org/10.1016/J.SCITOTENV.2021.148426.
(13) Wang, J.; Nabi, M. M.; Erfani, M.; Goharian, E.; Baalousha, M. Identification and Quantification of Anthropogenic Nanomaterials in Urban Rain and Runoff Using Single Particle-Inductively Coupled Plasma-Time of Flight-Mass Spectrometry. *Environ. Sci. Nano* **2022**, *9* (2), 714–729. https://doi.org/10.1039/D1EN00850A.
(14) Cedeño Laurent, J. G.; Parhizkar, H.; Calderon, L.; Lizonova, D.; Tsiodra, I.; Mihalopoulos, N.; Kavouras, I.; Alam, M.; Baalousha, M.; Bazina, L.; Kelesidis, G. A.; Demokritou, P. Physicochemical Characterization of the Particulate Matter in New Jersey/New York City Area, Resulting from the Canadian Quebec Wildfires in June 2023. *Environ. Sci. Technol.* **2024**, *58* (33), 14753–14763. https://doi.org/10.1021/acs.est.4c02016.
(15) Pace, H. E.; Rogers, N. J.; Jarolimek, C.; Coleman, V. A.; Higgins, C. P.; Ranville, J. F. Determining Transport Efficiency for the Purpose of Counting and Sizing Nanoparticles via Single Particle Inductively Coupled Plasma Mass Spectrometry. *Anal. Chem.* **2011**, *83* (24), 9361–9369. https://doi.org/10.1021/AC201952T/SUPPL_FILE/AC201952T_SI_001.PDF.
(16) Gundlach-Graham, A.; Hendriks, L.; Mehrabi, K.; Günther, D. Monte Carlo Simulation of Low-Count Signals in Time-of-Flight Mass Spectrometry and Its Application to Single-Particle Detection. *Anal. Chem.* **2018**, *90* (20), 11847–11855. https://doi.org/10.1021/ACS.ANALCHEM.8B01551/ASSET/IMAGES/LARGE/AC-2018-01551V_0007.JPEG.
(17) Rousseeuw, P. J. Silhouettes: A Graphical Aid to the Interpretation and Validation of Cluster Analysis. *J.*

Comput. Appl. Math. **1987**, *20* (C), 53–65. https://doi.org/10.1016/0377-0427(87)90125-7.
(18) Tsiodra, I.; Grivas, G.; Tavernaraki, K.; Paraskevopoulou, D.; Parinos, C.; Tsagkaraki, M.; Liakakou, E.; Bougiatioti, A.; Gerasopoulos, E.; Mihalopoulos, N. Profiling Aerosol Polycyclic Aromatic Compounds (PACs) in a Severely Polluted European City: A Comprehensive Assessment of the Residential Biomass Burning Impact on Atmospheric Toxicity. *J. Hazard. Mater.* **2025**, *494*, 138431. https://doi.org/10.1016/J.JHAZMAT.2025.138431.
(19) Cohen, J. M.; Beltran-Huarac, J.; Pyrgiotakis, G.; Demokritou, P. Effective Delivery of Sonication Energy to Fast Settling and Agglomerating Nanomaterial Suspensions for Cellular Studies: Implications for Stability, Particle Kinetics, Dosimetry and Toxicity. *NanoImpact* **2018**, *10*, 81–86. https://doi.org/10.1016/J.IMPACT.2017.12.002.
(20) DeLoid, G. M.; Cohen, J. M.; Pyrgiotakis, G.; Demokritou, P. Preparation, Characterization, and in Vitro Dosimetry of Dispersed, Engineered Nanomaterials. *Nat. Protoc.* **2017**, *12* (2), 355–371. https://doi.org/10.1038/nprot.2016.172.
(21) Deloid, G.; Cohen, J. M. J. M.; Darrah, T.; Derk, R.; Rojanasakul, L.; Pyrgiotakis, G.; Wohlleben, W.; Demokritou, P. Estimating the Effective Density of Engineered Nanomaterials for in Vitro Dosimetry. *Nat. Commun.* **2014**, *5*, 3514. https://doi.org/10.1038/ncomms4514.
(22) Smoke Smothers the Northeast https://earthobservatory.nasa.gov/images/151433/smoke-smothers-the-northeast. (accessed Jun 24, 2025).
(23) Smoke From Camp Fire Making Sacramento the Most Polluted City on Earth | Weather Underground https://www.wunderground.com/cat6/Smoke-Camp-Fire-Making-Sacramento-Most-Polluted-City-Earth (accessed Jun 24, 2025).
(24) Lizonova, D.; Nagarkar, A.; Demokritou, P.; Kelesidis, G. A. Effective Density of Inhaled Environmental and Engineered Nanoparticles and Its Impact on the Lung Deposition and Dosimetry. *Part. Fibre Toxicol.* **2024**, *21* (1), 7. https://doi.org/10.1186/s12989-024-00567-9.
(25) Ouf, F. X.; Bourrous, S.; Fauvel, S.; Kort, A.; Lintis, L.; Nuvoli, J.; Yon, J. True Density of Combustion Emitted Particles: A Comparison of Results Highlighting the Influence of the Organic Contents. *J. Aerosol Sci.* **2019**, *134*, 1–13. https://doi.org/10.1016/j.jaerosci.2019.04.007.
(26) DeLoid, G. M.; Cohen, J. M.; Pyrgiotakis, G.; Pirela, S. V.; Pal, A.; Liu, J.; Srebric, J.; Demokritou, P. Advanced Computational Modeling for in Vitro Nanomaterial Dosimetry. *Part. Fibre Toxicol.* **2015**, *12* (1), 32. https://doi.org/10.1186/s12989-015-0109-1.
(27) Bazina, L.; Deloid, G.; Fritzky, L.; Lizonova, D.; Vaze, N.; Demokritou, P. Impact of Canadian Wildfire-Emitted Particulate Matter on THP-1 Lung Macrophage Health and Function. *Environ. Sci. Technol.* **2025**, *59* (8). https://doi.org/10.1021/ACS.EST.4C10304.
(28) Daigneault, M.; Preston, J. a.; Marriott, H. M.; Whyte, M. K. B.; Dockrell, D. H. The Identification of Markers of Macrophage Differentiation in PMA-Stimulated THP-1 Cells and Monocyte-Derived Macrophages. *PLoS One* **2010**, *5* (1). https://doi.org/10.1371/journal.pone.0008668.
(29) DeLoid, G. M.; Sulahian, T. H.; Imrich, A.; Kobzik, L. Heterogeneity in Macrophage Phagocytosis of Staphylococcus Aureus Strains: High-Throughput Scanning Cytometry-Based Analysis. *PLoS One* **2009**, *4* (7).
(30) DeLoid, G.; Casella, B.; Pirela, S.; Filoramo, R.; Pyrgiotakis, G.; Demokritou, P.; Kobzik, L. Effects of Engineered Nanomaterial Exposure on Macrophage Innate Immune Function. *NanoImpact* **2016**, *2*, 70–81. https://doi.org/10.1016/j.impact.2016.07.001.
(31) Lansdon, L. A.; Cadieux-Dion, M.; Herriges, J. C.; Johnston, J.; Yoo, B.; Alaimo, J. T.; Thiffault, I.; Miller, N.; Cohen, A. S. A.; Repnikova, E. A.; Zhang, L.; Farooqi, M. S.; Farrow, E. G.; Saunders, C. J. Clinical Validation of Genome Reference Consortium Human Build 38 in a Laboratory Utilizing Next-Generation Sequencing Technologies. *Clin. Chem.* **2022**, *68* (9), 1177–1183. https://doi.org/10.1093/clinchem/hvac113.
(32) Das, M.; Calderon, L.; Singh, D.; Majumder, S.; Bazina, L.; Vaze, N.; Trivanovic, U.; DeLoid, G.; Zuverza-Mena, N.; Kaur, M.; Konkol, J.; Tittikpina, N. K.; Tsilomelekis, G.; Sadik, O.; White, J. C.;

Demokritou, P. Development and Characterization of Reference Environmentally Relevant Micro-Nano-Plastics for Risk Assessment Studies. *NanoImpact* **2025**, *38*, 100567. https://doi.org/10.1016/J.IMPACT.2025.100567.
